# An HIV-Tat inducible mouse model system of childhood HIV-associated nephropathy

**DOI:** 10.1242/dmm.045641

**Published:** 2020-10-28

**Authors:** Pingtao Tang, Jharna R. Das, Jinliang Li, Jing Yu, Patricio E. Ray

**Affiliations:** 1 Center for Genetic Medicine Research, Children's National Hospital, Washington, DC 20010, USA; 2 Department of Pediatrics, The George Washington University School of Medicine, Washington, DC 20052, USA; 3 Child Health Research Center, Department of Pediatrics, University of Virginia School of Medicine, Charlottesville, VA 22908, USA

**Keywords:** HIV-associated nephropathy, HIV-1, Children, Transgenic mice, Animal model, HIV-Tat

## Abstract

Modern antiretroviral therapies (ART) have decreased the prevalence of HIV-associated nephropathy (HIVAN). Nonetheless, we continue to see children and adolescents with HIVAN all over the world. Furthermore, once HIVAN is established in children, it is difficult to revert its long-term progression, and we need better animal models of childhood HIVAN to test new treatments. To define whether the HIV-1 trans-activator (*Tat*) gene precipitates HIVAN in young mice, and to develop an inducible mouse model of childhood HIVAN, an HIV-Tat gene cloned from a child with HIVAN was used to generate recombinant adenoviral vectors (rAd-*Tat*). rAd-*Tat* and *LacZ* control vectors (2×10^9^) were expressed in the kidney of newborn wild-type and HIV-transgenic (Tg_26_) FVB/N mice without significant proteinuria (*n*=5; 8 per group). Mice were sacrificed 7 and 35 days later to assess their renal outcome, the expression of HIV-genes and growth factors, and markers of cell growth and differentiation by RT-qPCR, immunohistochemistry and/or western blots. HIV-Tat induced the expression of HIV-1 genes and heparin-binding growth factors in the kidney of HIV-Tg_26_ mice, and precipitated HIVAN in the first month of life. No significant renal changes were detected in wild-type mice infected with rAd-*Tat* vectors, suggesting that HIV-Tat alone does not induce renal disease. This new mouse model of childhood HIVAN highlights the critical role that HIV-Tat plays in the pathogenesis of HIVAN, and could be used to study the pathogenesis and treatment of HIVAN in children and adolescents.

## Abstract (precis)

**Summary:** The development of a new inducible mouse model system of childhood HIV-associated nephropathy demonstrated that HIV-Tat plays a critical role in this disease, acting in synergy with other HIV-1 genes and heparin-binding cytokines.

## INTRODUCTION

Modern combined antiretroviral therapies (cART) have improved the clinical outcome in children and adolescents living with HIV, and have decreased the prevalence of HIV-associated nephropathy (HIVAN) to a significant degree. However, physicians have had less success providing chronic cART to children and adolescents living with HIV, and we continue to see HIVAN in this group of people all over the world. Over 80% of the estimated 2.1 million HIV-infected children are living in Sub-Saharan Africa (Mcculloch and Ray, 2008; Ray et al., 1998) and it is anticipated that ∼10% of these children will develop HIVAN if they do not receive appropriate ART (Mcculloch and Ray, 2008). Furthermore, we have noticed that once the typical renal histological features of HIVAN are established in children, it is difficult to prevent its long-term progression to end-stage kidney disease (ESKD) with current available treatments. In addition, previous reports in adults (Lescure et al., 2012; Izzedine et al., 2005) and children (Hegde et al., 2011) suggest that HIVAN can occur in people with suppressed viral load. These studies suggest that inflammatory cytokines released by HIV-infected cells can play a role in the pathogenesis of HIVAN independently of the viral load. Taken together, all of these findings underscore the importance of acquiring a better understanding of the pathogenesis and treatment of childhood HIVAN during the modern cART era.

Childhood HIVAN is a renal disease seen predominantly in Black children and adolescents who acquired HIV-1 through vertical transmission and do not receive appropriate ART (Strauss et al., 1989a; Ray et al., 1998). From the clinical point of view it is characterized by persistent proteinuria, often in the nephrotic range, and in the late stages it is associated with edema, reduced GFR, hypertension and rapid progression to ESKD (Strauss et al., 1989a; Ray et al., 1998; Mcculloch and Ray, 2008). The renal histological lesions of childhood HIVAN reveal mesangial hyperplasia, focal segmental or collapsing glomerulosclerosis, and multicystic tubular dilatation leading to renal enlargement (Strauss et al., 1989a; Ray et al., 1998; Mcculloch and Ray, 2008).

Several HIV-transgenic (HIV-Tg) animal models are available to study the pathogenesis and treatment of HIVAN (Dickie et al., 1991; Kopp et al., 1994; Ray et al., 2003; Zhong et al., 2005; Rosenstiel et al., 2009). However, these animals develop HIVAN at different time points, usually after they reach adulthood, and we lack a reliable mouse model system to study the pathogenesis of childhood HIVAN. Therefore, we carried out this study to determine whether the HIV-1 transactivator (*Tat*) gene precipitates HIVAN in young mice, and define whether this approach could be used to generate an inducible mouse model system of childhood HIVAN. To accomplish this goal, we infected newborn wild-type and heterozygous HIV-Tg_26_ mice with recombinant adenoviral vectors (rAd) carrying the coding sequence of the HIV-transactivator gene (HIV-Tat), and assessed the renal outcome of these mice during the first month of life.

## RESULTS

### Expression of HIV-Tat derived from a child with HIVAN in the kidney of newborn mice

**Fig. 1.**
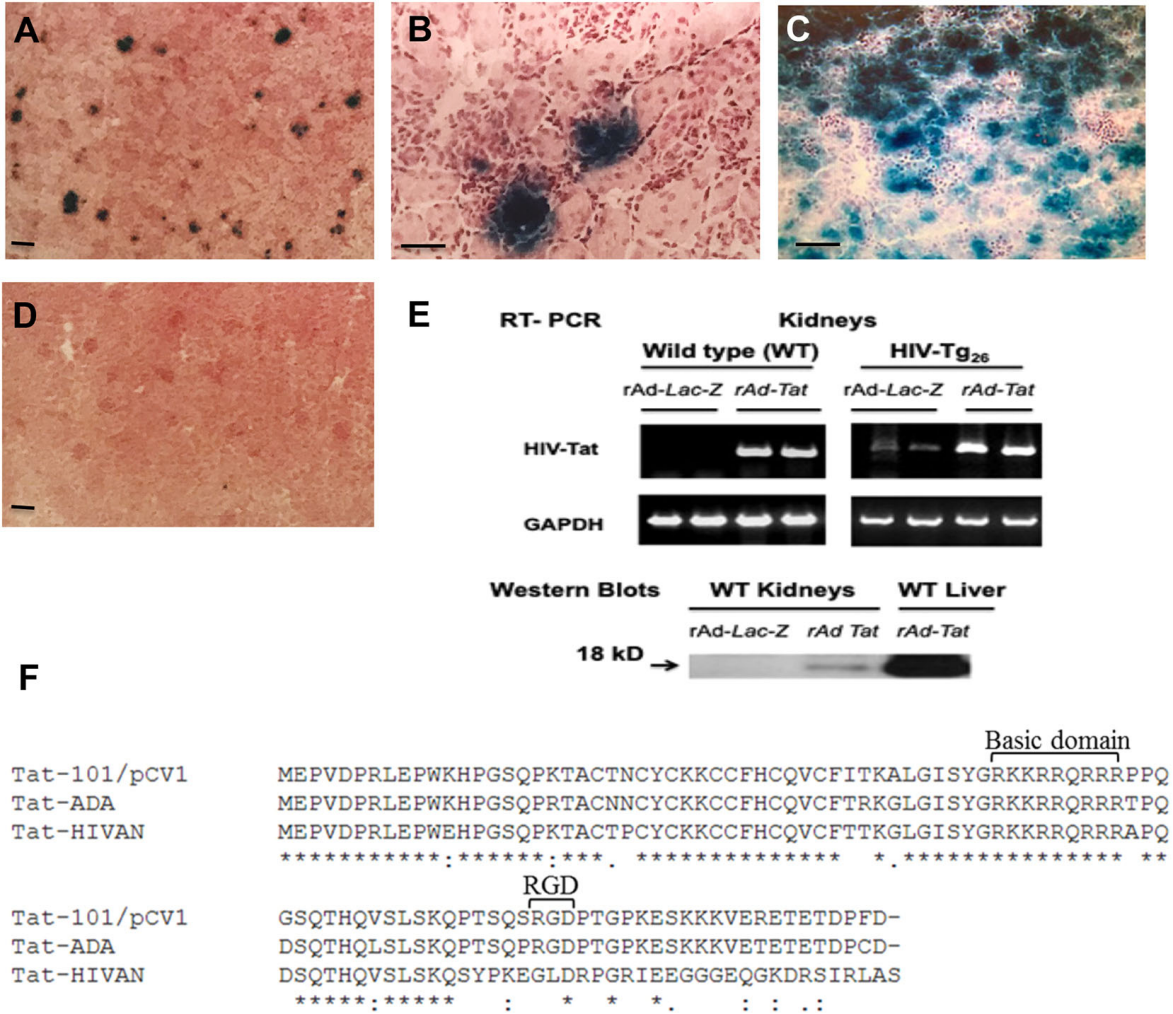
**LacZ** **and HIV-Tat expression in the kidneys** **of newborn wild-type and HIV-Tg_26_ mice infected with adenoviral vectors carrying LacZ or the HIV-Tat coding sequences.** (A-C) Adenovirus-mediated LacZ expression (blue color) in the renal cortex (A,B) and liver (C) of newborn mice injected with rAd-*LacZ* vectors (2×10^9^ particles per gram). All mice were euthanized 48 h after the injection. (D) No LacZ staining was observed in the renal cortex of an adult mouse injected with rAd-*LacZ* vectors and euthanized 48 h after the injection. (E) Representative RT-PCR results corresponding to HIV-Tat mRNA expression in the kidney of young wild-type (WT) and HIV-Tg_26_ mice infected either with rAd-*LacZ* or rAd-*Tat* vectors. Seven days after the adenoviral injections, all mice were sacrificed and their kidneys harvested and processed for the RT-PCR studies using specific Tat primers, as described in Materials and Methods. The lower panel shows western blots for HIV-Tat protein expression in kidney and liver tissues from wild-type mice infected with rAd-*LacZ* or rAd-*Tat* vectors. (F) Protein sequence of the HIV-Tat gene derived from a child with HIVAN aligned with HIV-Tat derived from the lymphotropic HIV-IIIB virus or the monocyte-tropic HIV-1 virus ADA, using the Clustal Omega multiple sequence alignment program. The basic domain and RGD motifs are indicated in brackets. The Tat sequencing procedure was repeated three times to rule out the possibility of a sequencing error. Scale bars: 50 μm.

Using an adenovirus gene transferring technique developed in our laboratory (Jerebtsova et al., 2005), we were able to express the *Escherichia coli lacZ* gene encoding β-galactosidase and the HIV-Tat gene in renal glomeruli of wild-type and HIV-Tg_26_ newborn mice (Fig. 1A,B,E). As expected, *LacZ* and Tat were also expressed in the liver of newborn mice (Fig. 1C-E). rAd-*Tat* vectors induced the renal expression of the HIV-Tg_26_ transgene, which carries a non-infectious 7.4 kb proviral HIV genome (pNL4-3) lacking 3 kb of sequence overlapping the *gag* and *pol* genes, but containing the 5′ and 3′ long terminal repeats (LTR) and all other HIV-accessory genes (Felser et al., 1989; Dickie et al., 1991). HIV-Tg_26_ newborn mice infected with rAd-*Tat* showed higher kidney expression levels of Tat mRNA compared to transgenic mice injected with rAd-*LacZ* vectors (Fig. 1E). The Tat mRNA detected in HIV-Tg_26_ mice injected with rAd-*LacZ* vectors was transcribed from the HIV-transgene described above (Felser et al., 1989). Fig. 1F shows the protein sequence of the HIV-Tat used in this study, which was derived from a child with HIVAN (Tat-HIVAN), and aligned with Tat protein sequences derived from the lymphotropic virus HIV-1 IIIB and the monocyte-tropic HIV-1 virus ADA (National Institutes of Health AIDS Research and Reference Reagent Program). As shown in Fig. 1A, Tat-HIVAN contains the basic domain that is essential for HIV-1 activation but is missing the RGD motif that interacts with cytokines and integrin receptors (Barillari et al., 1999a,b).

### Tat*-*induced expression of HIV-genes, fibroblast growth factor-2 (Fgf2), and vascular endothelial growth factor (Vegfa) in young HIV-Tg_26_ mice

**Fig. 2.**
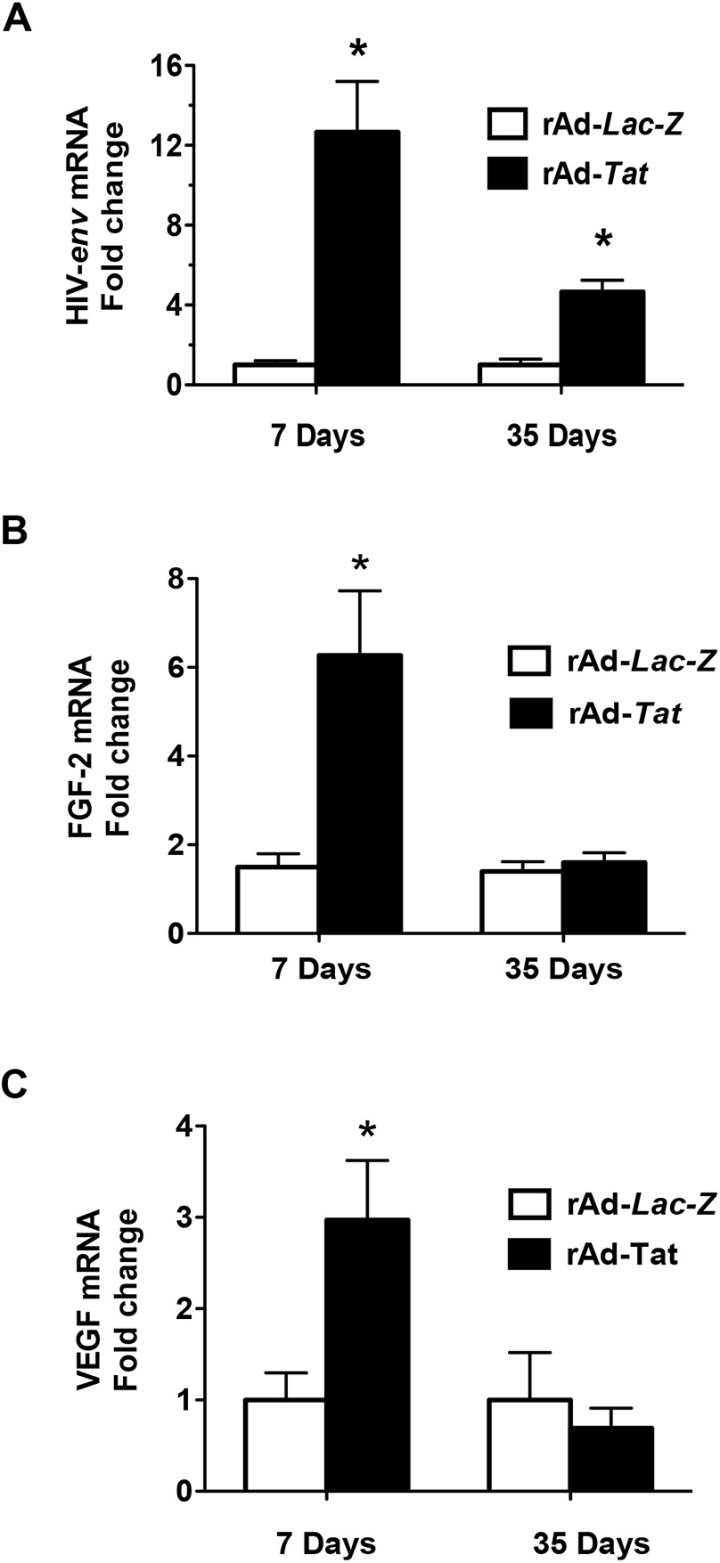
**rAd-*Tat* increased the expression of HIV-envelope (*env*), Fibroblast Growth Factor-2 (Fgf2) and Vascular Endothelial Growth Factor (Vegfa) mRNA.** (A-C) RNA was extracted from the kidney of 7- and 35-day-old HIV-Tg_26_ mice infected with rAd-*LacZ* or rAd-*Tat* vectors (*n*=4-6 mice per group). Real-time qRT-PCR analysis of HIV-*env* (A), FGF-2 (B) and VEGF (C) was performed as described in Materials and Methods. Data are mean±s.e.m. Statistical significance was determined using a Mann–Whitney unpaired *t*-test analysis between the two groups at 7 and 35 days of life. *P<0.05.

Seven-day-old HIV-Tg_26_ mice injected with rAd-*Tat* vectors showed higher renal expression levels of HIV-envelope (*env*) mRNA (∼12-fold) with RT-qPCR (Fig. 2A). RT-PCR also showed that the renal expression of HIV-*nef* and *rev* was also upregulated in 7-day-old HIV-Tg_26_ mice (∼sixfold) (Fig. S1). In addition, the mRNA expression levels of two heparin-binding cytokines (FGF-2 and VEGF-A) that are involved in the pathogenesis of HIVAN in HIV-Tg_26_ mice and children living with HIV (Ray et al., 1994; Korgaonkar et al., 2008; Soler-García et al., 2009), were elevated in the kidneys of 7-day-old HIV-Tg_26_ mice (Fig. 2B,C). Subsequently, the renal expression levels of HIV-*env* mRNA decreased over a period of days but continued to be elevated in 35-day-old HIV-Tg_26_ mice injected with rAd-*Tat* vectors (∼fivefold), compared to those injected with rAd-*LacZ* vectors (Fig. 2A). In contrast, lower expression levels of Fgf2, and Vegfa were noted at 35 days. The later findings are consistent with the immune-mediated clearance of the Tat adenoviral vectors.

### Tat*-*induced HIVAN in HIV-Tg_26_ mice

**Fig. 3.**
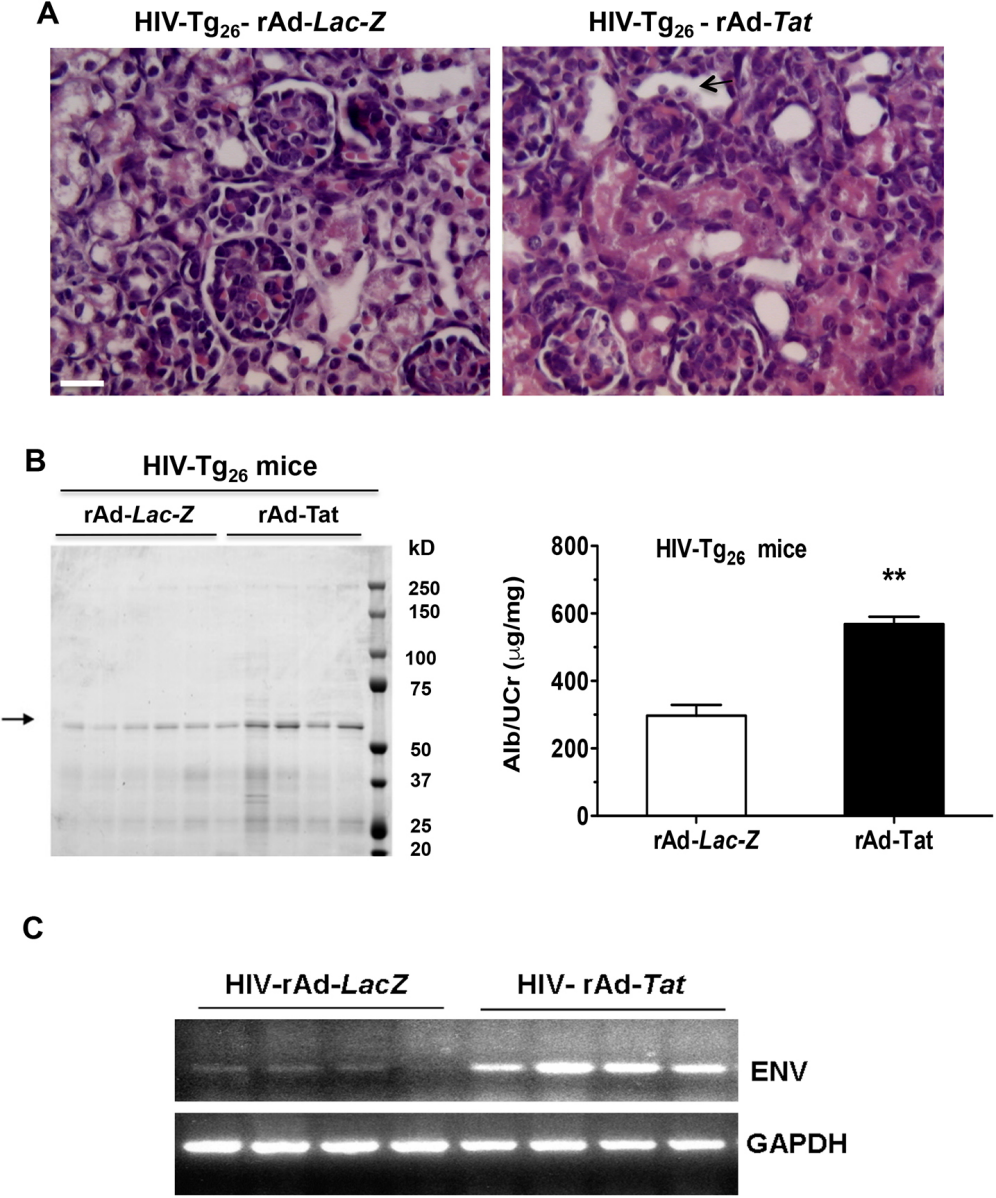
**rAd-*Tat* induced the expression of the HIV-envelope (*env*) gene in the kidneys of 7-day-old HIV-Tg_26_ mice, in association with the development of renal injury and albuminuria.** (A) Representative renal sections harvested from 7-day-old HIV-Tg_26_ mice injected either with rAd-*LacZ* or rAd-*Tat* vectors, and stained with hematoxylin and eosin. The black arrow points to injured tubular epithelial cells. Scale bar: 20 μm. (B) Coomassie blue-stained SDS-PAGE gel loaded with urine samples (5 µl) collected from 7-day-old HIV-Tg_26_ mice injected either with rAd-*LacZ* or rAd-*Tat* vectors (*n*=5 per group). The black arrow points to the albumin-stained bands. Albuminuria was quantified as described in Materials and Methods and expressed as a ratio of the urinary creatinine. (C) Expression of HIV-envelope (*env*) and GAPDH mRNA by RT-PCR in the kidney of 7-day-old HIV-Tg_26_ mice infected with either rAd-*LacZ* or rAd-*Tat* vectors (*n*=4 mice per group). Data are mean±s.e.m. Statistical significance was determined using a Mann–Whitney unpaired *t*-test (*n*=5 mice per group). **P<0.01.

**Fig. 4.**
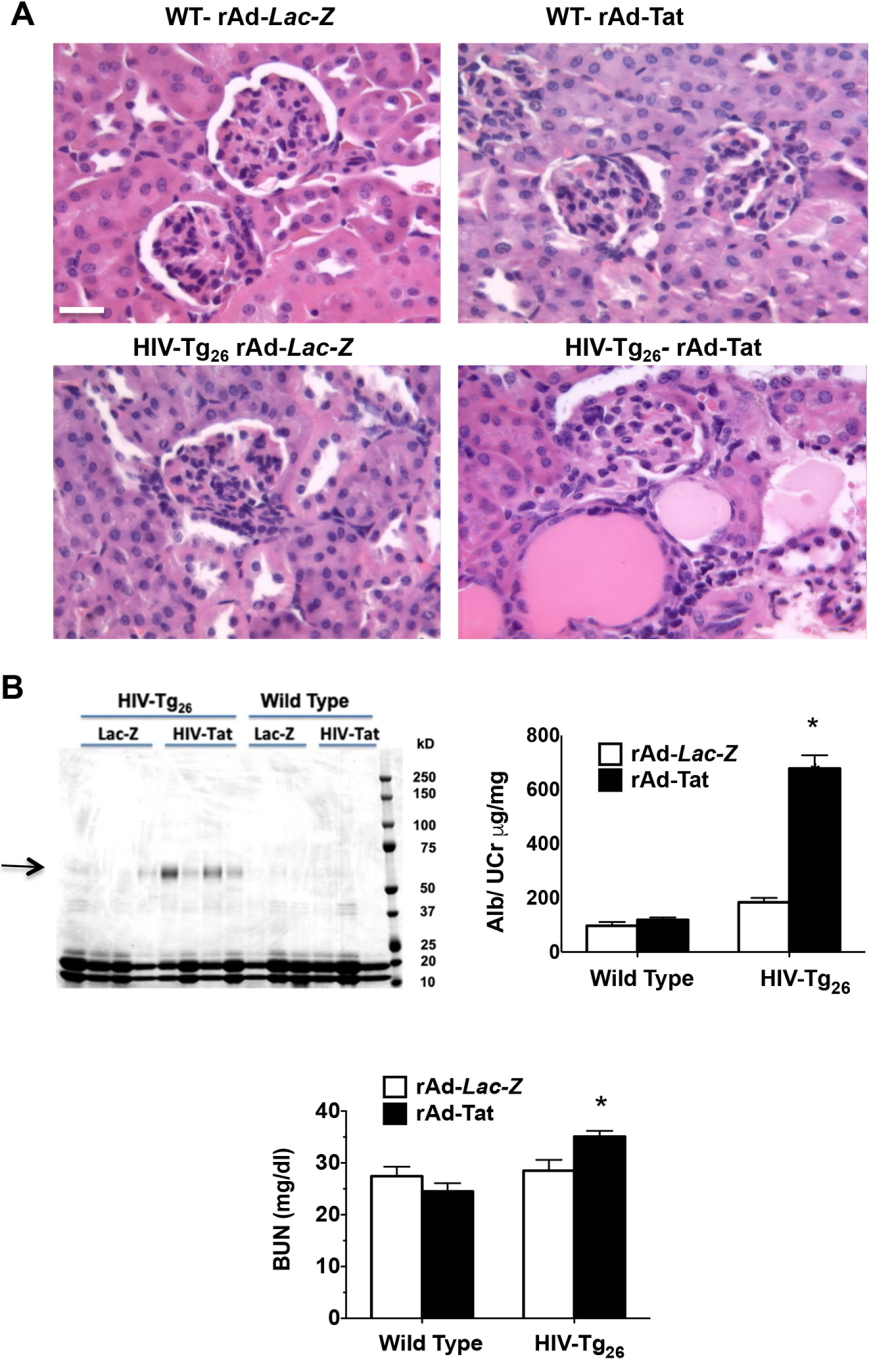
**rAd-*Tat* induced albuminuria and HIVAN in 35-day-old HIV-Tg_26_ mice.** (A) Representative renal sections harvested from 35-day-old wild-type (WT) and HIV-Tg_26_ mice infected with rAd-*LacZ* or rAd-*Tat* vectors, and stained with hematoxylin and eosin. Scale bar: 20 μm. (B) Coomassie blue-stained SDS-PAGE gel loaded with urine samples (5 microliters) collected from 35-day-old wild-type and HIV-Tg_26_ mice infected with either rAd-*LacZ* or rAd-*Tat* vectors (*n*=3-4 per group). *HIV-*Tg_26_ mice infected with rAd-*Tat* vectors developed significant albuminuria (black arrow, left panel), reported as a ratio of the urinary creatinine concentration (right panel; *P<0.006; ANOVA, *n*=4-5 mice per group). The BUN levels were elevated only in HIV-Tg_26_ mice infected with rAd-*Tat* vectors (bottom panel; *P<0.04; ANOVA, *n*=4-5 mice per group). Data are mean±s.e.m.

Seven-day-old HIV-Tg_26_ mice injected with rAd-*Tat* developed proteinuria and renal histological injury in association with an upregulated expression of HIV-*env* (Fig. 3A-C). Furthermore, 35 days after the rAd-*Tat* injections, HIV-Tg_26_ mice developed more significant HIVAN-like lesions, compared to the HIV-Tg_26_ mice injected with rAd-*LacZ* vectors. The HIVAN renal injury scores of 35-day-old HIV-Tg_26_ mice injected with rAd-*Tat* or rAd-*LacZ* were 3.16±0.30* versus 1.66±0.33, mean±s.e.m., respectively (*P=0.02, Mann–Whitney test; *n*=6 per group) (Fig. 4A,B). Furthermore, the blood urea nitrogen (BUN) levels were elevated only in HIV-Tg_26_ mice injected with the rAd-*Tat* vectors (Fig. 4B). In contrast, no significant differences in renal injury scores were noted between wild-type mice injected with rAd-LacZ and rAd-*Tat* vectors at 35 days of life (0.40±0.24 versus 0.80±0.37, mean±s.e.m., respectively; *P*=0.27, Mann–Whitney test; *n*=5 per group) (Fig. 4A,B). Overall, these findings suggest that Tat plays an important role precipitating HIVAN in HIV-Tg_26_ mice.

### Renal proliferative and apoptotic changes in young HIV-Tg_26_ mice

**Fig. 5.**
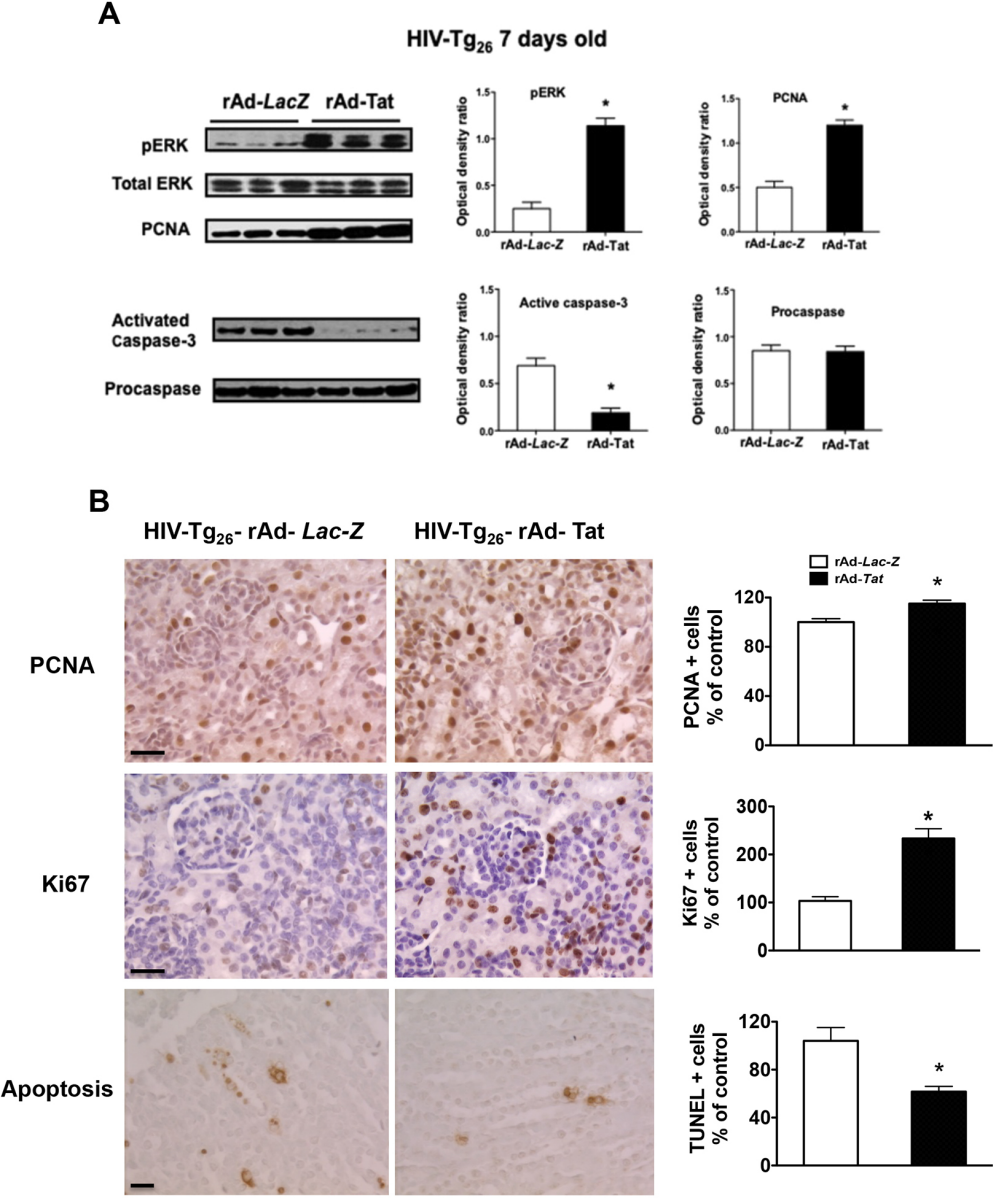
**rAd-*Tat* induced proliferative and anti-apoptotic changes in the kidneys of 7-day-old HIV-Tg_26_ mice.** (A) Representative results of the western blot analysis for pERK, PCNA, activated caspase 3, and procaspase performed with kidney homogenates derived from 7-day-old HIV-Tg_26_ mice infected with rAd-*Tat* or rAd-*LacZ* vectors (*n*=5 mice per group). The expression of PCNA was quantified as a ratio of β-actin. The graphs show the results of the densitometry analysis and quantification of the results in optical density units (mean±s.e.m.), as described in Materials and Methods. (B) Representative immunohistochemistry staining for PCNA, Ki-67 antigen, and apoptosis assessed using a TUNEL assay (all brown color), in renal sections harvested from 7-day-old HIV-Tg_26_ mice infected with either rAd-*Tat* or rAd-*LacZ* vectors. The graphs represent percentage changes in positive cells (mean±s.e.m.) relative to controls (*P<0.05, Mann–Whitney *t*-test, compared to HIV-Tg_26_ mice infected with the rAd-*LacZ* control vectors; *n*=4-5 per group). Statistical significance was determined using a Mann–Whitney unpaired *t*-test. *P<0.05. Scale bars: 20 μm.

**Fig. 6.**
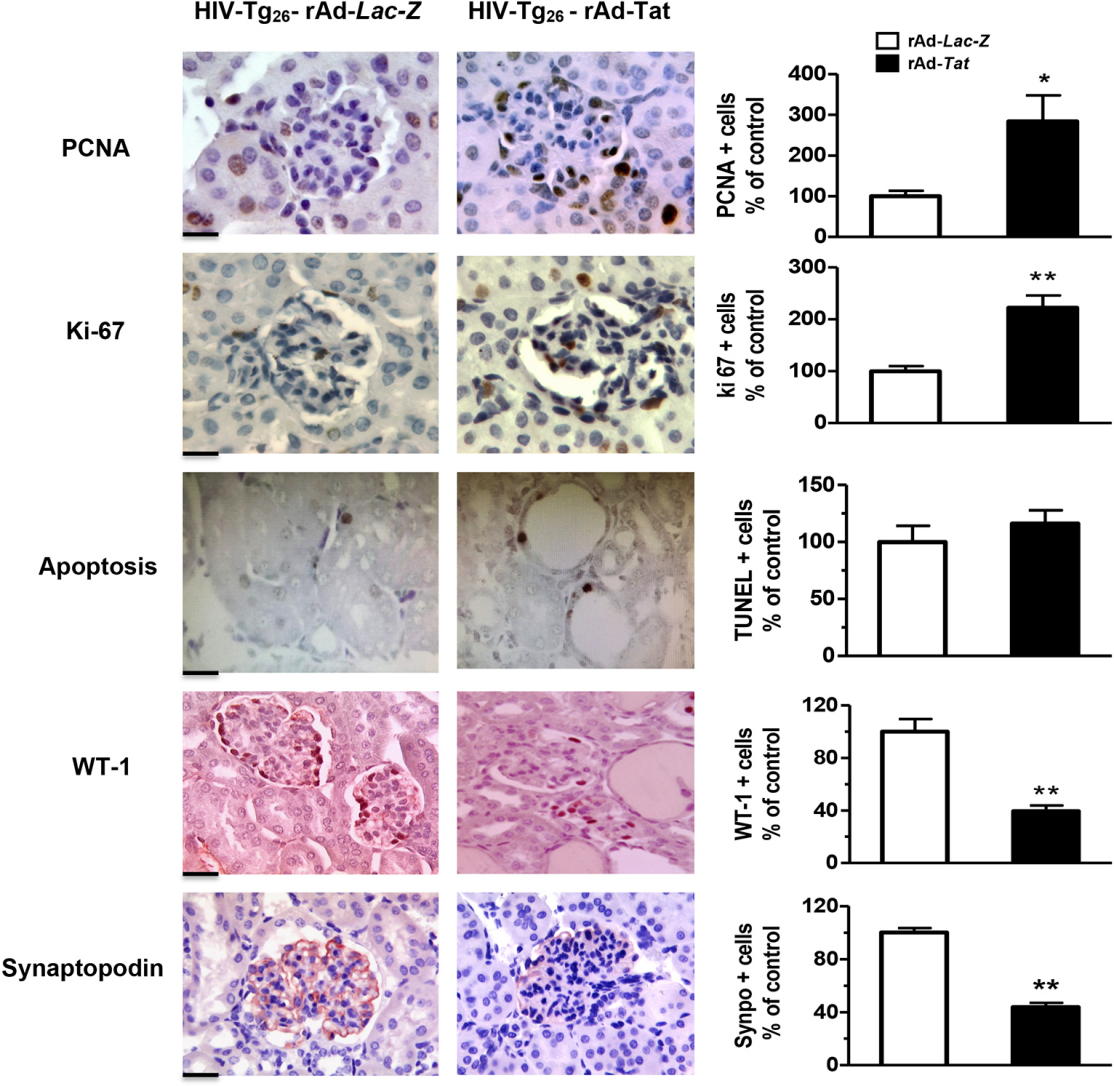
**rAd-*Tat* induced proliferative and dedifferentiation changes in podocytes of 35**-**day-old HIV-Tg_26_ mice*.*** The panels show representative immunohistochemistry staining for the PCNA, Ki-67 antigen, apoptosis assessed by a TUNEL assay (all brown color), WT1 antigen (red color), and synaptopodin (red color) in renal sections harvested from 35-day-old HIV-Tg_26_ mice infected with either rAd-*Tat* or rAd-*LacZ* vectors. The graphs represent percentage changes in positive cells per field (mean±s.e.m.) relative to the controls compared to the HIV-Tg_26_ mice infected with the rAd-*LacZ* control vectors (*P<0.05 or **P<0.01, Mann–Whitney *t*-test, *n*=4-5 per group). Data are mean±s.e.m. Statistical significance was determined using a Mann–Whitney unpaired *t*-test. *P<0.05, **P<0.01. Scale bars: 20 μm.

**Fig. 7.**
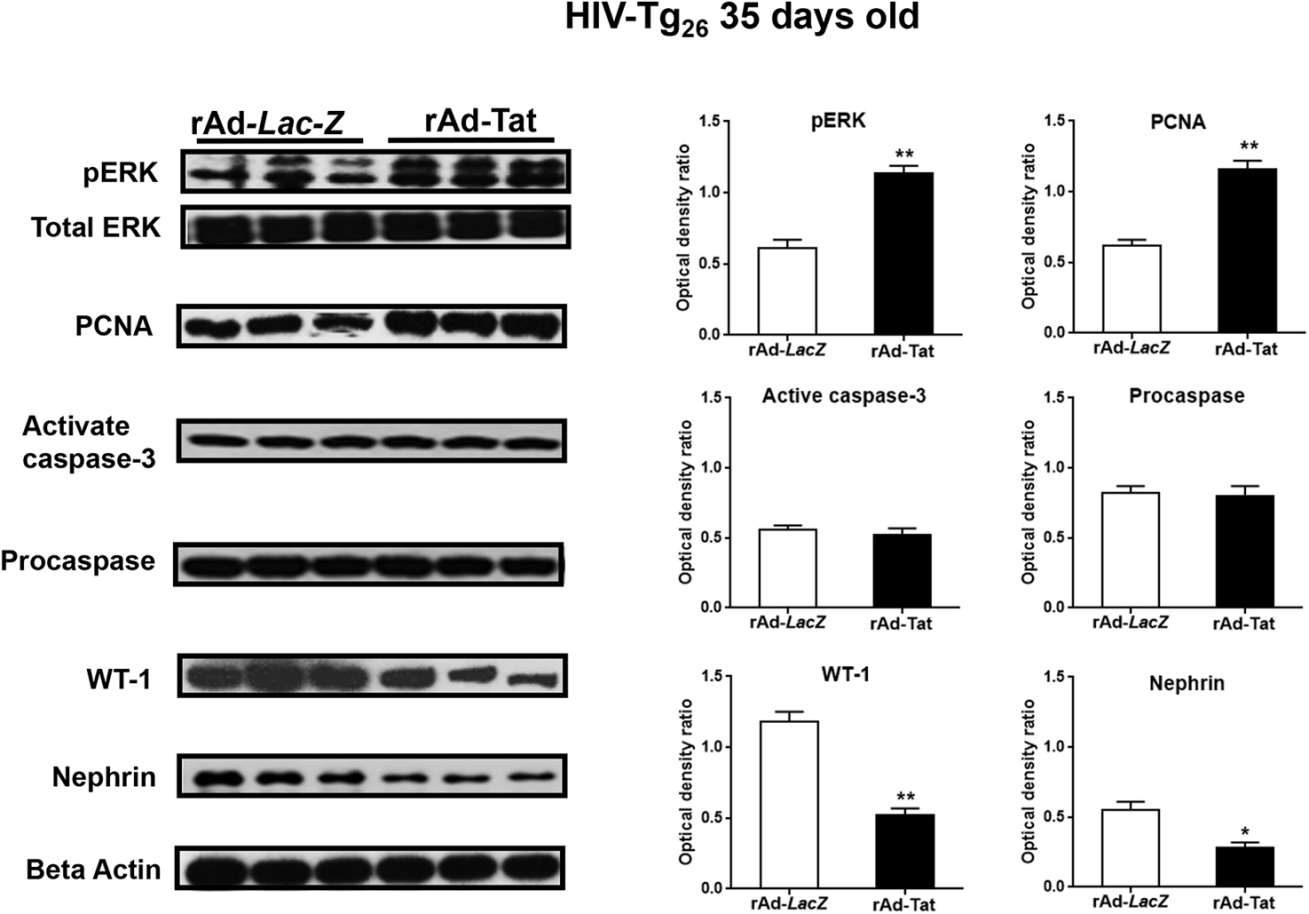
**Representative western blot analysis of the proliferative and dedifferentiation changes seen in the kidneys of 35**-**day-old HIV-Tg_26_ mice infected with rAd-*Tat* vectors.** Representative western blot analysis for pERK, PCNA, activated caspase 3, procaspase, WT1 and nephrin carried out with kidney homogenates derived from 35-day-old HIV-Tg_26_ mice infected with rAd-*Tat* or rAd-*LacZ* vectors (*n*=5 mice per group). The expression of PCNA, WT1 and nephrin was quantified using arbitrary optical density units expressed as a ratio of β-actin. The graphs show the results of the densitometry analysis and quantification of the results in optical density units (mean±s.e.m.), as described in Materials and Methods. Statistical significance was determined using a Mann–Whitney unpaired *t*-test. *P<0.05, **P<0.01. *n*=5 mice per group.

**Fig. 8.**
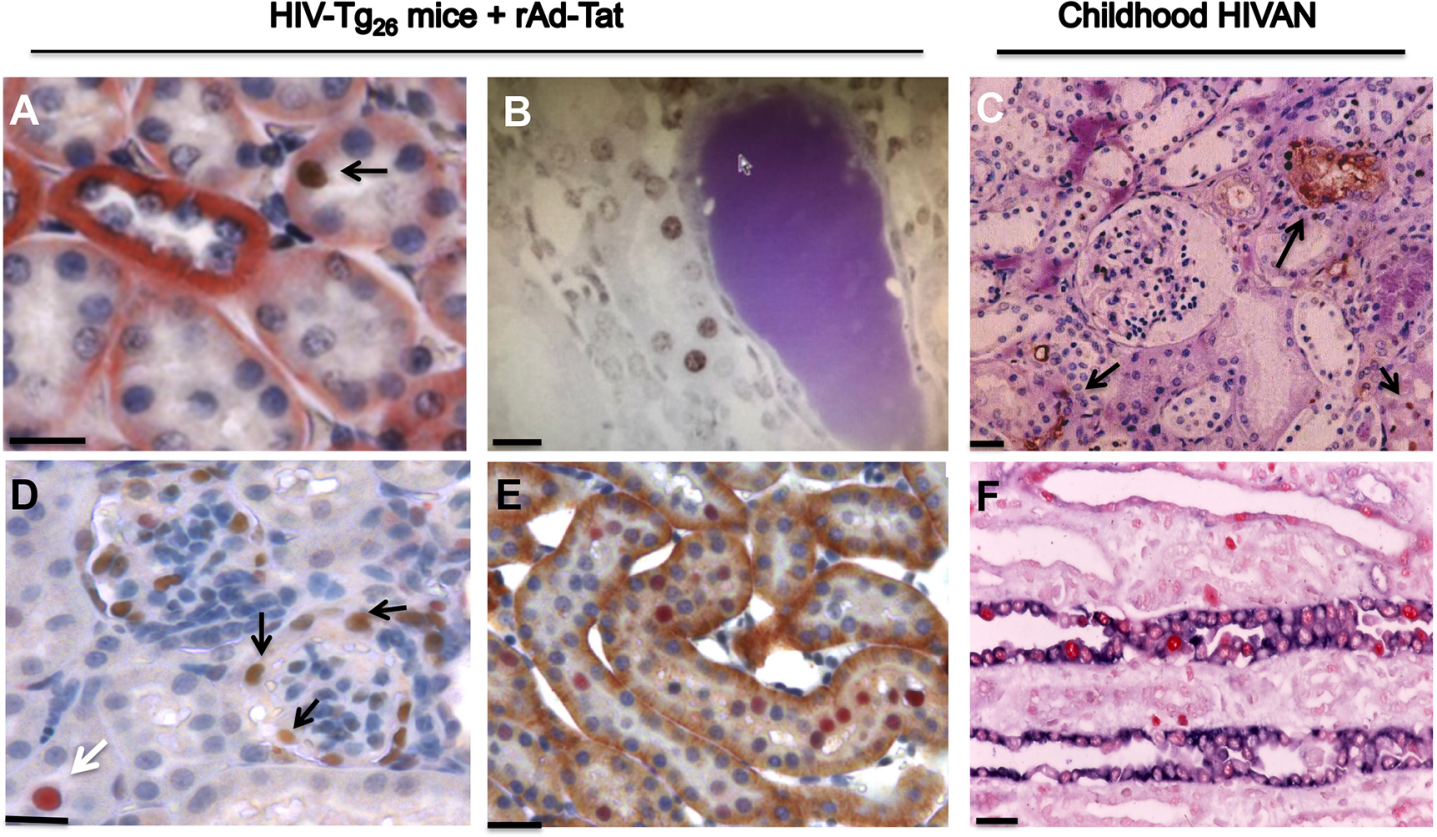
**Apoptosis and proliferative changes in the kidneys of HIV-Tg_26_ mice and children with HIVAN.** (A) Tubular epithelial cells stained red with an antibody against the Na^+^/K^+^ ATPase. The black arrow points to a renal tubular epithelial cell stained brown with a TUNEL assay for apoptosis. (B) Dilated renal tubules (arrowhead) from an HIV-Tg_26_ mouse infected with the rAd-*Tat* vector. Several epithelial cells are stained brown with a TUNEL assay for apoptosis. (C) Representative kidney section from a young child with HIVAN showing several tubular epithelial cells stained brown with a TUNEL assay for apoptosis (black arrows). (D) Renal cortex of an HIV-Tg_26_ mouse co-stained with antibodies against WT1 (brown color) and PCNA (red color). The black arrows point to cells that express both WT1 and PCNA antigens (orange color). The white arrow points to a tubular epithelial cell stained red with the PNCA antibody. (E) Renal section from an HIV-Tg_26_ mouse co-stained with antibodies against Na^+^/K^+^ ATPase (brown color) and PCNA (red color). (F) Renal section from a child with HIVAN co-stained with antibodies against pan-cytokeratin (violet color) and PCNA (red color). Scale bars: 20 μm (A,B,D-F); 10 μm (C).

HIV-Tg_26_ mice injected with rAd-*Tat* developed significant renal epithelial proliferative changes. Briefly, immunohistochemistry and western blot studies revealed that the expression levels of proliferating cell nuclear antigen (PCNA), Ki-67, and phospho-p44/42 MAPK (pERK) were elevated both in 7- and 35-day-old HIV-Tg_26_ mice injected with rAd-*Tat*, compared to those injected with rAd-*LacZ* vectors (Figs 5–7). In contrast, no changes were detected in the kidneys of 7- or 35-day-old wild-type mice injected with rAd-*Tat* or *LacZ* vectors (Figs S2, S3). Taken together, these findings suggest that HIV-Tat interacts with other HIV-1 genes and/or cytokines to induce the proliferation of renal epithelial cells (Korgaonkar et al., 2008; Ray et al., 1994; Ensoli et al., 1994). Alternatively, using the *in situ* TUNEL assay for apoptosis, as well as western blots to detect caspase 3 activation, we found a reduced number of renal epithelial cells undergoing apoptosis in 7-day-old HIV-Tg_26_ mice injected with rAd-*Tat*, relative to those infected with the control rAd-*LacZ* vectors (Fig. 5A,B). In contrast, no significant differences in apoptosis or caspase 3 activation were noted between 35-day-old HIV-Tg_26_ mice injected with rAd-*LacZ* or Tat vectors (Figs 6, 7). A more in-depth histological assessment of the renal sections revealed that apoptosis was detected predominately in tubular epithelial cells of 7-day-old HIV-Tg_26_ mice (Fig. 5), dilated tubular structures in 35-day-old HIV-Tg_26_ mice and young children with HIVAN (Fig. 6, Fig. 8A-C). In contrast, very few apoptotic changes were noted in glomerular epithelial cells (Fig. 8A-C, Fig. S4). Instead, as shown in children with HIVAN, a large number of proliferating cells were noted in glomerular and tubular epithelial cells of HIV-Tg_26_ mice injected with rAd-*Tat* vectors (Fig. 8D-F).

### Dedifferentiation of podocytes in young HIV-Tg_26_ mice

Immunohistochemistry and western blot studies carried out in kidney sections derived from 7-day-old HIV-Tg_26_ mice infected with rAd-*Tat* and LacZ vectors showed no significant differences in the expression levels of the podocyte-specific proteins nephrin, WT1 and synaptopodin (Fig. S5). However, the protein expression levels of nephrin, WT1 and synaptopodin were significantly reduced in 35-day-old HIV-Tg_26_ mice injected with rAd-*Tat* vectors (Figs 6–7). In summary, by 35 days of life, almost all HIV-Tg_26_ mice infected with rAd-HIV-Tat vectors develop all clinical and renal histological features consistent with childhood HIVAN (Kopp et al., 1992; Ray et al., 1994; Mcculloch and Ray, 2008) (Fig. 8).

## DISCUSSION

In this study we describe a new inducible mouse model system of childhood HIVAN. This model mimics the physiological process by which HIV-1 transcription is activated in humans and reproduces the full HIVAN phenotype in young HIV-Tg_26_ mice. Our findings underscore the critical role that HIV-Tat plays in the pathogenesis of HIVAN by inducing the renal expression of HIV-1 genes in synergy with heparin-binding growth factors and by increasing the dedifferentiation and proliferation of renal epithelial cells.

To develop the mouse model of childhood HIVAN, we took advantage of the HIV-Tg_26_ mouse line (Dickie et al., 1991; Kopp et al., 1992; Ray et al., 1994). These mice carry a 7.4 kb HIV-1 construct lacking a 3 kb sequence overlapping the *gag/pol* region of the HIV-provirus pNL4-3 (Felser et al., 1989), and express HIV-1 transcripts in many tissues, including kidney glomerular and tubular epithelial cells (Dickie et al., 1991; Kopp et al., 1992; Ray et al., 1994). Homozygous HIV-Tg_26_ mice are born sick and usually die with multiple systemic lesions during the first days or weeks of life (Dickie et al., 1991; Kopp et al., 1992). In contrast, heterozygous mice can be followed until they reach adulthood and have been used by several investigators to explore the pathogenesis of HIVAN (Dickie et al., 1991; Kopp et al., 1992; Korgaonkar et al., 2008; Ray et al., 1994). Because the majority of heterozygous HIV-Tg_26_ mice develop HIVAN at different time points after they reach adulthood, currently we do not have a reliable mouse model system to study the pathogenesis of childhood HIVAN. Therefore, we carried out this study to test the hypothesis that the induction of HIV-genes in the kidney of newborn mice precipitates HIVAN during the first month of life. To accomplish this goal, we used an adenovirus gene transferring technique, developed in our laboratory, that is based on the principle that the retention of adenoviral vectors in the circulation improves the transduction of renal glomerular cells in rodents (Ye et al., 2000, 2001). In previous studies, we showed that newborn mice have a delayed clearance of rAd vectors from the circulation, as well as an increased number of coxsackie-adenovirus receptors in glomerular cells (Jerebtsova et al., 2005). Both factors lead to the more efficient transduction of newborn glomerular cells after a systemic injection of adenoviruses via the retro-orbital plexus (Jerebtsova et al., 2005). The *LacZ* staining observed in renal glomeruli of newborn mice within 48 h of viral injection supports this notion, as no staining was detected in adult mice treated in a similar manner. In earlier studies, we also found that glomerular podocytes and endothelial cells are more susceptible to the adenoviral transduction compared to mesangial cells (Ye et al., 2002). Therefore, in our mouse model, podocytes and glomerular endothelial cells are more likely to be infected by the adenoviral vectors. Following this approach, we expressed the coding sequences of a Tat gene derived from a child with HIVAN (Tat-HIVAN) in the kidney of newborn HIV-Tg_26_ mice, and precipitated the development of HIVAN during the first month of life. Our findings support the results of previous studies showing that HIV-1 genes expressed in the kidney play a critical role in this process, although we do not yet understand the exact mechanisms involved. Further studies are warranted to explore this issue.

The HIV-Tat protein is a powerful transcriptional factor encoded by two exons. The first exon encodes the HIV activation and basic binding domains, which are required for HIV-transcription and nuclear localization of Tat (Hauber et al., 1989). The second exon encodes the RGD motif (C-terminal amino acids 73–86), which enhances the angiogenic activity of Tat acting through cytokines and integrin receptors (Boykins et al., 1999). Tat plays an essential role in HIV-replication by recruiting a cellular human protein called cyclin T1, which efficiently increases the transcription of the HIV-LTR via NF-κB (Berkhout et al., 1989). However, cloning and characterization of the murine CycT1 protein revealed that mouse cyclin 1 lacks a critical cysteine residue that is needed to form a complex with Tat and induce its full transcriptional activity (Garber et al., 1998; Bieniasz et al., 1998). For this reason, Tat has limited direct transcriptional activity in mice but it can induce the expression of TNF-α (Chen et al., 1997) and other cytokines that increase the transcription of HIV-1 via NF-κB-dependent mechanisms (Ben Haij et al., 2015). Our study showed that the activation and basic binding domains of Tat are sufficient to induce the renal expression of HIV-genes and precipitate HIVAN in young mice. In contrast, we found that the RGD motif of Tat is not essential in this process.

In addition to being a powerful activator of HIV-1 transcription, Tat is released into the circulation by infected cells and can be taken up by uninfected cells (Barillari et al., 1999b; Ensoli et al., 1994). In adult mice, previous studies showed that the immune attack on *LacZ*-transduced hepatocytes releases the β-galactosidase protein produced by these cells in the circulation (Zhu et al., 1997). β-galactosidase is subsequently deposited in the kidney, resulting in the blue positive LacZ staining of glomerular cells (Zhu et al., 1997). However, this immune-related process requires a few days, as in our case no β-galactosidase protein produced in the liver was detected in the glomeruli of adult mice sacrificed 48 h after the adenoviral injection. Although the detection of Tat mRNA in the kidney, as well as the LacZ glomerular staining in newborn mice sacrificed within 48 h of the viral infection, demonstrates that glomerular cells were transduced by the adenoviral vectors, we cannot rule out the possibility that Tat released into the circulation by hepatocytes at later time points might be deposited in the kidney, thus contributing to the pathogenesis of HIVAN. In any case, children with HIV-renal diseases are also exposed to circulating Tat that can be accumulated in the kidney and endothelial cells bound to heparan sulfate proteoglycans (Ray et al., 2006; Urbinati et al., 2009). In this manner, Tat can mimic the action of several cytokines involved in the pathogenesis of AIDS, including SDF-1α, RANTES and MIF1-β (Barillari et al., 1999b; Ensoli et al., 1994; Buonaguro et al., 1992). Furthermore, acting in synergy with FGF-2, Tat can induce the dedifferentiation and proliferation of cultured human podocytes (Conaldi et al., 2002; Xie et al., 2014; Das et al., 2016). For these reasons, we explored the effects of Tat in wild-type mice but were unable to detect significant renal lesions in these mice. Our findings suggest that Tat alone cannot induce renal disease in wild-type mice. However, we should mention that Tat-HIVAN has an incomplete RGD sequence; therefore, its ability to interact with cytokines and integrin receptors *in vivo* might be impaired (Barillari et al., 1999a,b). A previous study showed that a full-length Tat-alone expressed specifically in podocytes did not induce renal disease in adult mice (Zuo et al., 2006). However, further studies are needed to determine whether Tat proteins containing RDG sequences, which can be released in the circulation and stored bound to heparan sulfate proteoglycans located on the cell surface of other kidney cell types (Ray et al., 2006), can cause kidney damage in young mice. We speculate that a potential mechanism through which Tat could precipitate HIVAN in HIV-Tg_26_ mice is by increasing the production and/or activity of TNF-α (Chen et al., 1997), given that high levels of TNF-α are detected in the circulation of HIV-Tg_26_ mice (De et al., 2002) and that TNF-α worsens the outcome of HIVAN in adult mice (Bruggeman et al., 2011). Alternatively, Tat could act in synergy with FGF-2 and VEGF-A (Ben Haij et al., 2015; Ray et al., 1994; Korgaonkar et al., 2008; Xie et al., 2014), as both heparin-binding growth factors were upregulated by Tat in 7-day-old HIV-Tg_26_ mice and have been linked to the pathogenesis of HIVAN in children and HIV-Tg_26_ mice (Ray et al., 1994, 2006; Korgaonkar et al., 2008). Finally, Tat also induced the renal expression of *nef* in young HIV-Tg_26_ mice, and *nef* plays a critical role in the pathogenesis of HIVAN in mice (Zhong et al., 2005; Rosenstiel et al., 2009).

Overall, our mouse model reproduces all the renal histological features characteristic of childhood HIVAN (Strauss et al., 1989b; Mcculloch and Ray, 2008). Interestingly, the expression levels of the podocyte-specific proteins, nephrin, WT1, and synaptopodin, did not change in correlation with the induction of HIV-1 genes during the first week of life. These findings suggest that the podocyte dedifferentiation changes characteristic of HIVAN might be a secondary event associated with the regeneration of these cells. It is possible that podocytes that express high levels of HIV-1 genes die and are replaced by parietal epithelial or renal progenitor cells (Dijkman et al., 2006; Ronconi et al., 2009), which do not express podocytes markers and are sensitive to the growth promoting effects of several growth factors (Barasch et al., 1997). In addition, we noted a reduced number of renal epithelial cells undergoing apoptosis in 7-day-old HIV-Tg_26_ mice infected with rAd-*Tat* vectors compared to the controls. It is tempting to speculate that Tat, in combination with bFGF-2 and VEGF-A, might have an anti-apoptotic effect (Sgadari et al., 2011), as both heparin-binding growth factors were upregulated at this time point. However, as shown in children with HIVAN, apoptotic changes were also detected in dilated renal tubules of 35-day-old HIV-Tg_26_ mice infected with rAd-*Tat* vectors. In contrast, very few apoptotic cells were detected in glomerular epithelial cells. Therefore, the process of apoptosis does not appear to play a major role in inducing the dedifferentiation of podocytes. Finally, we found that Tat induced glomerular and tubular epithelial proliferative changes in 7- and 35-day-old HIV-Tg_26_ mice. These changes appear to be driven by the pERK signaling pathway, which can be activated directly by HIV-Nef (Lu et al., 2008; Sunamoto et al., 2003; He et al., 2004; Snyder et al., 2010), as well as FGF-2 (Ray et al., 1994) or VEGF-A (Korgaonkar et al., 2008). In summary, by the end of the first month of life, all HIV-Tg_26_ mice infected with rAd-*Tat* vectors develop proteinuria and renal histological lesions consistent with those seen in children with HIVAN.

In humans, the risk variants of apolipoprotein-1 (APOL1) increase the lifetime risk of untreated HIV^+^ people developing HIVAN by ∼50% (Genovese et al., 2010; Kopp and Winkler, 2003; Kasembeli et al., 2015). Therefore, one limitation of our animal model is that HIV-Tg_26_ mice do not express the APOL-1 gene. This limitation could be overcome by generating dual transgenic HIV-Tg_26_/APOL1 mice (Bruggeman et al., 2016) and infecting newborn mice with rAd-*Tat* vectors. In addition, a significant number of Black children living with HIV develop HIVAN independently of the APOL1 risk variants (Ng et al., 2017; Purswani et al., 2016), and previous studies suggest that the APOL1 risk variants might play a more relevant role in adults compared to young children (Purswani et al., 2016; Woroniecki et al., 2016). Thus, young kidneys might be more sensitive to the cytotoxic effects of HIV-1 genes, TNF-α and heparin-binding growth factors, and less dependent on the APOL1 risk variants to develop HIVAN. Alternatively, it is possible that other unknown genetic factors might play an additional role in precipitating HIVAN in Black children, as is the case with HIV-Tg_26_ mice that are known to carry an HIVAN susceptibility locus on chromosome 3 (Gharavi et al., 2004). Taken together, these studies show that a strong genetic influence modulates the outcome of HIVAN in mice and humans, and more work needs to be done to define these factors in children.

In conclusion, we have developed an inducible mouse model system of childhood HIVAN that reproduces the full HIVAN phenotype during the first month of life. In addition, we showed that Tat plays a relevant role in this process by inducing the renal expression of HIV-1 genes, FGF-2, and VEGF-A, leading to the activation of pERK. Hopefully, this animal model will facilitate the discovery of new therapeutic targets to prevent the progression of HIVAN in children and adolescents.

## MATERIALS AND METHODS

### Experimental design

This study was approved by the Children's Research Institute Animal Care and Use Committee. We used HIV-Tg_26_ transgenic mice (Dickie et al., 1991) carrying a 7.4-kb-long non-infectious clone of the pNL4-3 provirus lacking a 3 kb sequence overlapping the gag and pol genes but including the 5′ and 3′ LTRs and the *env*, *tat*, *nef*, *rev*, *vif*, *vpr* and *vpu* HIV-genes (Felser et al., 1989). Heterozygous newborn HIV-Tg_26_ FVB/N mice (Dickie et al., 1991; Kopp et al., 1992) and their wild-type littermates were injected through the retro-orbital plexus with 2×10^9^ particle-to-plaque-forming unit (pfu)/mouse of rAd vectors carrying either HIV-Tat derived from a child with HIVAN (rAd-*Tat* vector) or the *E*. *coli LacZ* gene (rAd-*LacZ*). To express the HIV-Tat rAd vector in the kidney of newborn mice, we used a gene transferring technique developed in our laboratory (Jerebtsova et al., 2005). Wild-type and HIV-Tg_26_ mice were divided into groups (*n*=8 mice each) and sacrificed 7 days (peak of rAd-*Tat* expression) and 35 days (renal clearance of the viral vectors) after the adenoviral injections. All mice had free access to water and standard food, and were treated in accordance with the National Institutes of Health guidelines for care and use of research animals.

### Adenoviral vectors

The generation of the rAd-*Tat* vector derived from a child with HIVAN has been described in detail previously (Xie et al., 2014). Briefly, a cDNA fragment encoding the full-length Tat protein was cloned into the pCXN2-FLAG vector and used to generate E1-deleted recombinant adenoviruses carrying Tat-HIVAN-FLAG (Xie et al., 2014). The protein sequence of the Tat-HIVAN gene was aligned and compared to other Tat genes using the Clustal Omega multiple sequence alignment program (www.ebi.ac.uk/Tools/msa/clustalo/). Both Tat-FLAG and *LacZ* control adenoviruses were amplified, purified, desalted and titrated as described previously (Kozarsky et al., 1993; Jerebtsova et al., 2005, 2007). The pfu ratio of the virus stock used in these experiments was 100. To detect the localization of β-galactosidase (LacZ) expression, frozen tissue sections (10 μm) were fixed in 0.5% glutaraldehyde (Sigma-Aldrich) at room temperature for 10 min, washed with PBS and stained for 2 h at 37°C in PBS containing 5 mM K3 Fe(CN)6, 5 mM K4 Fe(CN)6, 1 mM MgCl_2_ (all from Sigma-Aldrich), and 1 mg/ml 5-bromo-4-chloro-3-indolyl-β-D-galactopyranoside (X-gal, Boehringer Mannheim). The sections were then counterstained with hematoxylin (Fisher Scientific) and mounted for microscopic evaluation.

### Blood, urine and kidney sample collection

Mice were sacrificed 7 and 35 days after the rAd injections. Urine, blood and kidney samples were harvested and kept frozen at −80°C. Blood urea nitrogen was assessed using a QuantiChrom Urea Assay kit (BioAssay Systems, DIUR-500) as described previously (Mattison et al., 2012). The urinary creatinine levels were measured using a colorimetric assay (R&D Systems, KGE005). Albuminuria was measured with a mouse albumin ELISA kit (Bethyl Laboratories, E99-134) and expressed as ratio of the urinary creatinine. In addition, SDS-PAGE (4-12%) was carried out with 5 ml of urine that was then stained with Coomassie blue stain solution (Bio-Rad) to detect changes in high and low molecular weight urinary proteins as described previously (Das et al., 2016).

### Renal injury score

Each kidney cross-section was evaluated using a microscope with 20× and 40× magnification lenses. An average of 50 glomerular and tubular sections were assessed per group. The following parameters were used to develop a renal injury score: (1) the percentage of glomeruli exhibiting segmental or global sclerosis; (2) the percentage of tubular cast and microcysts; (3) the percentage of glomerular and tubular cells that stained positive for proliferating cell nuclear antigen (PCNA); (4) the percentage of glomerular and tubular cells that stained positive for apoptosis with a TUNEL assay; and (5) the percentage of glomerular showing decreased WT1 staining in podocytes. The mean values of all these results were added to generate the current renal injury score: 0=<5%; 1=5-10%; 2=11-25%; 3=26-50%; and 4=>50%.

### RT-PCR analysis

Total kidney RNA was isolated using TRIzol (Invitrogen, 15596-026) and treated with deoxyribonuclease I, following Invitrogen's protocol for RT-PCR studies. cDNA was generated from 3 µg RNA using the SuperScript III First-Strand Synthesis System for RT-PCR (Invitrogen, 18080-051). Tat mRNA expression was assessed by RT-PCR using the following primers: forward 5′-ATGGAGCCAGTAGATCCTAGAC-3′ and reverse 5′-CTAATCGAATCGATCTGTCTCTGC-3′. To determine the relative expression of HIV-1 envelope (*env*), we used the following primers: forward primer 5′-TGTGTAAAATTAACCCCACTCTG-3′ and reverse primer 5′-ACAACTTATCAACCTATAGCTGGT-3′. As a control, we amplified the mouse housekeeping gene glyceraldehyde-3-phosphate dehydrogenase (*Gapdh*) using the forward primer 5′-CTTACTCCTTGGAGGCCATGT-3′ and the reverse primer 5′-GCCAAGGTCATCCATGACAAC-3′. During the amplification process, samples were kept at 94°C for 4 min, followed by 35 cycles at 94°C for 30 s, 55°C for 30 s and 72°C for 1 min, and a final extension of 8 min at 72°C. For each HIV-envelope and *Gapdh* PCR amplification reaction, we used 5 µl and 2 µl of cDNA, respectively. The densitometry analysis was conducted using Adobe Photoshop 6.0, as described previously (Li et al., 2017; Xie et al., 2014).

### Real-time RT-PCR analysis

Real-time RT-PCR studies were performed on cDNA samples using a Platinum qPCR SuperMix-UDG kit (Invitrogen, 11730-017). The HIV-envelope assay was designed to amplify a 95-bp amplicon from HIV-1 NL4-3 (GenBank accession number AF324493) [forward primer 5′-CCTTTGAGCCAATTCCCATACATT-3′, reverse primer 5′-gacgttTGGTCCTGTTCCATTGAACGTC-3′ with fluorescein amidite (FAM)-labeled LUX]. The mouse nephrin assay was designed to amplify a 79-bp amplicon (GenBank accession number NM_019459.2) (forward primer 5′-GTCGGAGGAGGATCGAATCAG-3′, reverse primer 5′-cgggGTGGAGCTTCTTGTGTCCCG-3′ with FAM-labeled LUX). The mouse fibroblast growth factor 2 (*Fgf2*) assay was designed to amplify a 70-bp amplicon (GenBank accession number NM_008006) [forward primer 5′-CCGGTCACGGAAATACTCCAG-3′, reverse primer 5′-cgaactCCGAGTTTATACTGCCCAGTTCG-3′ with FAM-labeled LUX (Invitrogen, 19450335)]. The mouse vascular endothelial growth factor (VEGF_164_ isoform) assay was designed to amplify a 101-bp amplicon (GenBank accession number M95200.1) (forward primer 5′-cggcCTACCAGCGAAGCTACTGCCG-3′ with FAM-labeled LUX, reverse primer 5′-CACACAGGACGGCTTGAAGATG-3′). The mouse *Gapdh* housekeeping gene qPCR control assay was designed to amplify a 93-bp amplicon from (GenBank accession number NM_008084.1) (forward primer 5′-gacatacAGGCCGGTGCTGAGTATGT-3′ with JOE-labeled LUX, reverse primer 5′-TTTGGCTCCACCCTTCAAGT-3′). The real-time PCR amplification protocol was as follows: 50°C for 2 min hold (uracil-DNA glycosylase treatment); 95°C for 2 min; and 40 cycles of 95°C for 15 s, 58°C for 30 s, and 72°C for 30 s, using a 7900 Fast Real-Time PCR System (Applied Biosystems). Data were normalized to *Gapdh* and presented as fold increase compared to the rAd-*LacZ* control group. Real-time PCR amplification of the HIV-genes *nef* and *rev* was performed with the GoTaq qPCR Master Mix (Promega) using the US/Art7 primers (Klotman et al., 1991). These primers predominately amplify a PCR product of 203 bp representing the *nef* cDNA and a 219/255 bp PCR product representing the *rev* cDNA in HIV-Tg_26_ mice (Bruggeman et al., 1994). The PCR conditions were 95°C for 3 min, followed by 40 cycles of 95°C for 15 s, 55°C for 30 s and 60°C for 1 min. PCR products were resolved on 3% agarose gels.

### Western blot analysis

The kidneys were lysed using RIPA lysis buffer containing protease inhibitors and phosphatase inhibitor cocktail 2 (Sigma-Aldrich), and processed by western blots as described previously (Mattison et al., 2012). The following primary antibodies were used: phospho-p44/42 mitogen-activated protein kinase (Thr202/Tyr204), p44/42 mitogen-activated protein kinase ERK1/2 (both obtained from Cell Signaling Technology; 9101 and 9102, respectively), PCNA (C-20) rabbit polyclonal, β-actin (I-19) goat polyclonal, caspase-3 (pro and activate forms) rabbit polyclonal antibodies (Santa Cruz Biotechnology; sc-9857, sc-1616 and sc-7148, respectively), Wilms Tumor 1 (WT1) mouse monoclonal anti-human antibody clone 6F-H2 (Dako, M3561) and nephrin guinea pig polyclonal antibody (Progen Biotechnik, GP-N2). All primary antibodies were diluted 1:1000 except for WT1, which was diluted 1:500 and incubated overnight at 4°C. Protein bands were detected using Supersignal West Pico Chemiluminescent Substrate (Thermo Scientific) according to the manufacturer's instruction. All membranes were exposed to Kodak film (X-OMAT) and developed using an automated developer. Densitometry analysis of the data expressed as a β-actin ratio was conducted using Adobe Photoshop 6.0 as described previously (Xie et al., 2014).

### Immunohistochemistry

Paraffin-embedded sections (5 μm) were deparaffinized, rehydrated and stained as described previously (Jerebtsova et al., 2007). Immunostaining was performed with a commercial streptavidin-biotin-peroxidase complex (Histostain SP kit, Zymed) according to the manufacturer's instructions as described previously (Tang et al., 2005). The peroxidase activity was monitored after the addition of substrate using a DAB kit (Vector Laboratories, SK-4100) or AEC substrate kit (Invitrogen, 002007). Sections were counterstained with hematoxylin. The PCNA staining kit (Invitrogen, 931143) was used to detect PCNA. Ki-67 and WT1 staining was assessed using a 1:50 dilution of a monoclonal rat anti-mouse Ki-67 antibody (clone TEC-3, M7249) and a mouse monoclonal anti-human WT1 antibody (clone 6F-H2), respectively (both from Dako). Synaptopodin was detected with a ready-to-use mouse monoclonal antibody (clone G1D4, Batch number 1372) from Fitzgerald Industries International (10R-2373). Controls included replacing the primary antibody with equivalent concentrations of the corresponding nonspecific antibodies and/or omitting the first or second antibodies. Apoptosis was assessed using an ApopTag *in situ* apoptosis detection kit (Chemicon International, S7101) according to the manufacturer's instructions. Three renal sections from young children (<12 years of age) with HIVAN were obtained from archived autopsies performed at the Children's National Hospital, and stained with an ApopTag *in situ* apoptosis detection kit, as well as the anti-PCNA (PC 10) and pan-cytokeratin (AE1-AE3) antibodies (Dako; M8079 and M3515, respectively), to determine which cells show the most significant apoptotic and proliferative changes in children with HIVAN. The latter studies were approved by the Institutional Review Board of the Children's National Hospital with a waiver of consent. Double immunostaining for PCNA, Na^+^, K^+^, ATPase and WT1 in mouse tissues was performed with a M.O.M. (Mouse on Mouse) ImmPRESS horseradish peroxidase (HRP) Polymer kit (Vector Laboratories, MP-2400) and the ImmPRESS HRP horse anti-rabbit IgG (peroxidase) Polymer Detection Kit (Vector Laboratories, MP-7401). Primary antibodies included the mouse monoclonal anti-Na^+^/K^+^ ATPase antibody (1:10, Developmental Studies Hybridoma Bank, a6F), rabbit monoclonal anti-PCNA antibody (1:1000, Cell Signaling Technology, 13110) and mouse monoclonal anti-WT1 antibody Clone 6F-H2 (1:600, MilliporeSigma, MAB4234). Double staining for apoptosis and Na^+^/K^+^ ATPase was conducted using an ApopTag Peroxidase *In Situ* Apoptosis Detection kit (MilliporeSigma, S7100) according to the manufacturer's instructions, followed by the inactivation of HRP with 1% H_2_O_2_ in PBS and 0.1 M glycine (pH 2.2), and immunostaining for Na^+^/K^+^ ATPase with the M.O.M. (Mouse on Mouse) ImmPRESS HRP (Peroxidase) Polymer kit (Vector Laboratories, MP-2400).

### Statistical analysis

If not specified otherwise, the data were expressed as mean±s.e.m. Differences between two groups were compared using an unpaired two-tailed Student's *t-*test. Multiple sets of data were compared by ANOVA with Newman–Keuls post-hoc comparisons. Statistical analyses were performed using GraphPad Prism software (version 5.00; GraphPad Software). Values of *P*<0.05 were considered statistically significant.

## Supplementary Material

Supplementary information

## References

[DMM045641C1] Barasch J., Qiao J., McWilliams G., Chen D., Oliver J. A. and Herzlinger D. (1997). Ureteric bud cells secrete multiple factors, including bFGF, which rescue renal progenitors from apoptosis. *Am. J. Physiol.* 273, F757-F767. 10.1152/ajprenal.1997.273.5.F757 9374839

[DMM045641C2] Barillari G., Sgadari C., Fiorelli V., Samaniego F., Colombini S., Manzari V., Modesti A., Nair B. C., Cafaro A., Sturzl M. et al. (1999a). The Tat protein of Human Immunodeficiency Virus type-1 promotes vascular cell growth and locomotion by engaging the alpha5beta1 and alphavbeta3 integrins and by mobilizing sequestered basic Fibroblast Growth Factor. *Blood* 94, 663-672. 10397733

[DMM045641C3] Barillari G., Sgadari C., Palladino C., Gendelman R., Caputo A., Morris C. B., Nair B. C., Markham P., Nel A., Sturzl M. et al. (1999b). Inflammatory cytokines synergize with the HIV-1 Tat protein to promote angiogenesis and Kaposi's sarcoma via induction of basic Fibroblast Growth Factor and the alpha v beta 3 integrin. *J. Immunol.* 163, 1929-1935. 10438928

[DMM045641C4] Ben Haij N., Planès R., Leghmari K., Serrero M., Delobel P., Izopet J., BenMohamed L. and Bahraoui E. (2015). HIV-1 Tat protein induces production of proinflammatory cytokines by human dendritic cells and monocytes/macrophages through engagement of TLR4-MD2-CD14 complex and activation of NF-kappaB pathway. *PLoS ONE* 10, e0129425 10.1371/journal.pone.0129425 26090662 PMC4474861

[DMM045641C5] Berkhout B., Silverman R. H. and Jeang K.-T. (1989). Tat trans-activates the human immunodeficiency virus through a nascent RNA target. *Cell* 59, 273-282. 10.1016/0092-8674(89)90289-4 2478293

[DMM045641C6] Bieniasz P. D., Grdina T. A., Bogerd H. P. and Cullen B. R. (1998). Recruitment of a protein complex containing Tat and cyclin T1 to TAR governs the species specificity of HIV-1 Tat. *EMBO J.* 17, 7056-7065. 10.1093/emboj/17.23.7056 9843510 PMC1171053

[DMM045641C7] Boykins R. A., Mahieux R., Shankavaram U. T., Gho Y. S., Lee S. F., Hewlett I. K., Wahl L. M., Kleinman H. K., Brady J. N., Yamada K. M. et al. (1999). Cutting edge: a short polypeptide domain of HIV-1-Tat protein mediates pathogenesis. *J. Immunol.* 163, 15-20. 10384093

[DMM045641C8] Bruggeman L. A., Thomson M. M., Nelson P. J., Kopp J. B., Rappaport J., Klotman P. E. and Klotman M. E. (1994). Patterns of HIV-1 mRNA expression in transgenic mice are tissue-dependent. *Virology* 202, 940-948. 10.1006/viro.1994.1416 7518165

[DMM045641C9] Bruggeman L. A., Drawz P. E., Kahoud N., Lin K., Barisoni L. and Nelson P. J. (2011). TNFR2 interposes the proliferative and NF-kappaB-mediated inflammatory response by podocytes to TNF-alpha. *Lab. Invest.* 91, 413-425. 10.1038/labinvest.2010.199 21221075 PMC3075956

[DMM045641C10] Bruggeman L. A., Wu Z., Luo L., Madhavan S. M., Konieczkowski M., Drawz P. E., Thomas D. B., Barisoni L., Sedor J. R. and O'Toole J. F. (2016). APOL1-G0 or APOL1-G2 transgenic models develop preeclampsia but not kidney disease. *J. Am. Soc. Nephrol.* 27, 3600-3610. 10.1681/ASN.2015111220 27026370 PMC5118487

[DMM045641C11] Buonaguro L., Barillari G., Chang H. K., Bohan C. A., Kao V., Morgan R., Gallo R. C. and Ensoli B. (1992). Effects of the human immunodeficiency virus type 1 Tat protein on the expression of inflammatory cytokines. *J. Virol.* 66, 7159-7167. 10.1128/JVI.66.12.7159-7167.1992 1279199 PMC240407

[DMM045641C12] Chen P., Mayne M., Power C. and Nath A. (1997). The Tat protein of HIV-1 induces Tumor Necrosis Factor-alpha production. Implications for HIV-1-associated neurological diseases. *J. Biol. Chem.* 272, 22385-22388. 10.1074/jbc.272.36.22385 9278385

[DMM045641C13] Conaldi P. G., Bottelli A., Baj A., Serra C., Fiore L., Federico G., Bussolati B. and Camussi G. (2002). Human Immunodeficiency Virus-1 Tat induces hyperproliferation and dysregulation of renal glomerular epithelial cells. *Am. J. Pathol.* 161, 53-61. 10.1016/S0002-9440(10)64156-9 12107089 PMC1850697

[DMM045641C14] Das J. R., Gutkind J. S. and Ray P. E. (2016). Circulating fibroblast growth factor-2, HIV-Tat, and vascular endothelial cell growth factor-A in HIV-infected children with renal disease activate Rho-A and Src in cultured renal endothelial cells. *PLoS ONE* 11, e0153837 10.1371/journal.pone.0153837 27097314 PMC4838216

[DMM045641C15] De S. K., Devadas K. and Notkins A. L. (2002). Elevated levels of tumor necrosis factor alpha (TNF-alpha) in Human Immunodeficiency Virus type 1-transgenic mice: prevention of death by antibody to TNF-alpha. *J. Virol.* 76, 11710-11714. 10.1128/JVI.76.22.11710-11714.2002 12388730 PMC136749

[DMM045641C16] Dickie P., Felser J., Eckhaus M., Bryant J., Silver J., Marinos N. and Notkins A. L. (1991). HIV-associated nephropathy in transgenic mice expressing HIV-1 genes. *Virology* 185, 109-119. 10.1016/0042-6822(91)90759-5 1926769

[DMM045641C17] Dijkman H. B. P. M., Weening J. J., Smeets B., Verrijp K. C., van Kuppevelt T. H., Assmann K. K. J. M., Steenbergen E. J. and Wetzels J. F. M. (2006). Proliferating cells in HIV and pamidronate-associated collapsing focal segmental glomerulosclerosis are parietal epithelial cells. *Kidney Int.* 70, 338-344. 10.1038/sj.ki.5001574 16761013

[DMM045641C18] Ensoli B., Gendelman R., Markham P., Fiorelli V., Colombini S., Raffeld M., Cafaro A., Chang H.-K., Brady J. N. and Gallo R. C. (1994). Synergy between basic fibroblast growth factor and HIV-1 Tat protein in induction of Kaposi's sarcoma. *Nature* 371, 674-680. 10.1038/371674a0 7935812

[DMM045641C19] Felser J. M., Klimkait T. and Silver J. (1989). A syncytia assay for human immunodeficiency virus type I (HIV-I) envelope protein and its use in studying HIV-I mutations. *Virology* 170, 566-570. 10.1016/0042-6822(89)90448-0 2543131

[DMM045641C20] Garber M. E., Wei P., KewalRamani V. N., Mayall T. P., Herrmann C. H., Rice A. P., Littman D. R. and Jones K. A. (1998). The interaction between HIV-1 Tat and human cyclin T1 requires zinc and a critical cysteine residue that is not conserved in the murine CycT1 protein. *Genes Dev.* 12, 3512-3527. 10.1101/gad.12.22.3512 9832504 PMC317238

[DMM045641C21] Genovese G., Friedman D. J., Ross M. D., Lecordier L., Uzureau P., Freedman B. I., Bowden D. W., Langefeld C. D., Oleksyk T. K., Uscinski Knob A. L. et al. (2010). Association of trypanolytic ApoL1 variants with kidney disease in African Americans. *Science* 329, 841-845. 10.1126/science.1193032 20647424 PMC2980843

[DMM045641C22] Gharavi A. G., Ahmad T., Wong R. D., Hooshyar R., Vaughn J., Oller S., Frankel R. Z., Bruggeman L. A., D'Agati V. D., Klotman P. E. et al. (2004). Mapping a locus for susceptibility to HIV-1-associated nephropathy to mouse chromosome 3. *Proc. Natl. Acad. Sci. USA* 101, 2488-2493. 10.1073/pnas.0308649100 14983036 PMC356977

[DMM045641C23] Hauber J., Malim M. H. and Cullen B. R. (1989). Mutational analysis of the conserved basic domain of human immunodeficiency virus tat protein. *J. Virol.* 63, 1181-1187. 10.1128/JVI.63.3.1181-1187.1989 2536828 PMC247813

[DMM045641C24] He J. C., Husain M., Sunamoto M., D'Agati V. D., Klotman M. E., Iyengar R. and Klotman P. E. (2004). Nef stimulates proliferation of glomerular podocytes through activation of Src-dependent Stat3 and MAPK1,2 pathways. *J. Clin. Invest.* 114, 643-651. 10.1172/JCI200421004 15343382 PMC514582

[DMM045641C25] Hegde S., Singh C. and Ohare B. (2011). HIV-associated nephropathy in the setting of maximal virologic suppression. *Pediatr. Nephrol.* 26, 973-977. 10.1007/s00467-011-1783-3 21350798

[DMM045641C26] Izzedine H., Wirden M. and Launay-Vacher V. (2005). Viral load and HIV-associated nephropathy. *N. Engl. J. Med.* 353, 1072-1074. 10.1056/NEJMc051607 16148301

[DMM045641C27] Jerebtsova M., Liu X.-H., Ye X. and Ray P. E. (2005). Adenovirus-mediated gene transfer to glomerular cells in newborn mice. *Pediatr. Nephrol.* 20, 1395-1400. 10.1007/s00467-005-1882-0 16133067

[DMM045641C28] Jerebtsova M., Wong E., Przygodzki R., Tang P. and Ray P. E. (2007). A novel role of Fibroblast Growth Factor-2 and pentosan polysulfate in the pathogenesis of intestinal bleeding in mice. *Am. J. Physiol. Heart Circ. Physiol.* 292, H743-H750. 10.1152/ajpheart.00969.2006 17071728

[DMM045641C29] Kasembeli A. N., Duarte R., Ramsay M., Mosiane P., Dickens C., Dix-Peek T., Limou S., Sezgin E., Nelson G. W., Fogo A. B. et al. (2015). APOL1 risk variants are strongly associated with HIV-associated nephropathy in black South Africans. *J. Am. Soc. Nephrol.* 26, 2882-2890. 10.1681/ASN.2014050469 25788523 PMC4625661

[DMM045641C30] Klotman M. E., Kim S., Buchbinder A., DeRossi A., Baltimore D. and Wong-Staal F. (1991). Kinetics of expression of multiply spliced RNA in early human immunodeficiency virus type 1 infection of lymphocytes and monocytes. *Proc. Natl. Acad. Sci. UAS* 88, 5011-5015. 10.1073/pnas.88.11.5011 PMC51797 1711215

[DMM045641C31] Kopp J. B. and Winkler C. (2003). HIV-associated nephropathy in African Americans. *Kidney Int. Suppl.* 83, S43-S49. 10.1046/j.1523-1755.63.s83.39.x 12864874

[DMM045641C32] Kopp J. B., Klotman M. E., Adler S. H., Bruggeman L. A., Dickie P., Marinos N. J., Eckhaus M., Bryant J. L., Notkins A. L. and Klotman P. E. (1992). Progressive glomerulosclerosis and enhanced renal accumulation of basement membrane components in mice transgenic for human immunodeficiency virus type 1 genes. *Proc. Natl. Acad. Sci. USA* 89, 1577-1581. 10.1073/pnas.89.5.1577 1542649 PMC48495

[DMM045641C33] Kopp J. B., Ray P. E., Adler S. H., Bruggeman L. A., Mangurian C. V., Owens J. W., Eckhaus M. A., Bryant J. L. and Klotman P. E. (1994). Nephropathy in HIV-transgenic mice. *Contrib. Nephrol.* 107, 194-204. 10.1159/000422980 8004968

[DMM045641C34] Korgaonkar S. N., Feng X., Ross M. D., Lu T.-C., D'Agati V., Iyengar R., Klotman P. E. and He J. C. (2008). HIV-1 upregulates VEGF in podocytes. *J. Am. Soc. Nephrol.* 19, 877-883. 10.1681/ASN.2007050629 18443354 PMC2386717

[DMM045641C35] Kozarsky K., Grossman M. and Wilson J. M. (1993). Adenovirus-mediated correction of the genetic defect in hepatocytes from patients with familial hypercholesterolemia. *Somat. Cell Mol. Genet.* 19, 449-458. 10.1007/BF01233250 8291022

[DMM045641C36] Lescure F.-X., Flateau C., Pacanowski J., Brocheriou I., Rondeau E., Girard P.-M., Ronco P., Pialoux G. and Plaisier E. (2012). HIV-associated kidney glomerular diseases: changes with time and HAART. *Nephrol. Dial. Transplant.* 27, 2349-2355. 10.1093/ndt/gfr676 22248510

[DMM045641C37] Li J., Das J. R., Tang P., Han Z., Jaiswal J. K. and Ray P. E. (2017). Transmembrane TNF-alpha facilitates HIV-1 infection of podocytes cultured from children with HIV-associated nephropathy. *J. Am. Soc. Nephrol.* 28, 862-875. 10.1681/ASN.2016050564 27811066 PMC5328167

[DMM045641C38] Lu T. C., He J. C., Wang Z.-H., Feng X., Fukumi-Tominaga T., Chen N., Xu J., Iyengar R. and Klotman P. E. (2008). HIV-1 Nef disrupts the podocyte actin cytoskeleton by interacting with diaphanous interacting protein. *J. Biol. Chem.* 283, 8173-8182. 10.1074/jbc.M708920200 18234668 PMC2276381

[DMM045641C39] Mattison P. C., Soler-García A. A., Das J. R., Jerebtsova M., Perazzo S., Tang P. and Ray P. E. (2012). Role of circulating Fibroblast Growth Factor-2 in lipopolysaccharide-induced acute kidney injury in mice. *Pediatr. Nephrol.* 27, 469-483. 10.1007/s00467-011-2001-z 21959768 PMC3265667

[DMM045641C40] McCulloch M. I. and Ray P. E. (2008). Kidney disease in HIV-positive children. *Semin. Nephrol.* 28, 585-594. 10.1016/j.semnephrol.2008.09.001 19013330 PMC2778302

[DMM045641C41] Ng D. K., Robertson C. C., Woroniecki R. P., Limou S., Gillies C. E., Reidy K. J., Winkler C. A., Hingorani S., Gibson K. L., Hjorten R. et al. (2017). APOL1-associated glomerular disease among African-American children: a collaboration of the chronic kidney disease in children (CKiD) and nephrotic syndrome study network (NEPTUNE) cohorts. *Nephrol. Dial. Transplant.* 32, 983-990. 10.1093/ndt/gfw061 27190333 PMC5837652

[DMM045641C42] Purswani M. U., Patel K., Winkler C. A., Spector S. A., Hazra R., Seage G. R. III, Mofenson L., Karalius B., Scott G. B., Van Dyke R. B. et al. (2016). Brief report: APOL1 renal risk variants are associated with chronic kidney disease in children and youth with perinatal HIV infection. *J. Acquir. Immune Defic. Syndr.*, 73, 63-68. 10.1097/QAI.0000000000001010 27035887 PMC4981510

[DMM045641C43] Ray P. E., Bruggeman L. A., Weeks B. S., Kopp J. B., Bryant J. L., Owens J. W., Notkins A. L. and Klotman P. E. (1994). bFGF and its low affinity receptors in the pathogenesis of HIV-associated nephropathy in transgenic mice. *Kidney Int.* 46, 759-772. 10.1038/ki.1994.331 7996798

[DMM045641C44] Ray P. E., Rakusan T., Loechelt B. J., Selby D. M., Liu X. H. and Chandra R. S. (1998). Human Immunodeficiency Virus (HIV)-associated nephropathy in children from the Washington, D.C. area: 12 years’ experience. *Semin. Nephrol.* 18, 396-405. 9692352

[DMM045641C45] Ray P. E., Liu X.-H., Robinson L. R., Reid W., Xu L., Owens J. W., Jones O. D., Denaro F., Davis H. G. and Bryant J. L. (2003). A novel HIV-1 transgenic rat model of childhood HIV-1-associated nephropathy. *Kidney Int.* 63, 2242-2253. 10.1046/j.1523-1755.2003.00028.x 12753314

[DMM045641C46] Ray P. E., Tassi E., Liu X.-H. and Wellstein A. (2006). Role of Fibroblast growth factor-binding protein in the pathogenesis of HIV-associated hemolytic uremic syndrom. *Am. J. Physiol. Regul. Integr. Comp. Physiol.* 290, R105-R113. 10.1152/ajpregu.00492.2005 16352855

[DMM045641C47] Ronconi E., Sagrinati C., Angelotti M. L., Lazzeri E., Mazzinghi B., Ballerini L., Parente E., Becherucci F., Gacci M., Carini M. et al. (2009). Regeneration of glomerular podocytes by human renal progenitors. *J. Am. Soc. Nephrol.* 20, 322-332. 10.1681/ASN.2008070709 19092120 PMC2637058

[DMM045641C48] Rosenstiel P., Gharavi A., D'Agati V. and Klotman P. (2009). Transgenic and infectious animal models of HIV-associated nephropathy. *J. Am. Soc. Nephrol.* 20, 2296-2304. 10.1681/ASN.2008121230 19497967

[DMM045641C49] Sgadari C., Barillari G., Palladino C., Bellino S., Taddeo B., Toschi E. and Ensoli B. (2011). Fibroblast growth factor-2 and the HIV-1 tat protein synergize in promoting Bcl-2 expression and preventing endothelial cell apoptosis: implications for the pathogenesis of AIDS-associated Kaposi's sarcoma. *Int. J. Vasc. Med.* 2011, 452729 10.1155/2011/452729 22007303 PMC3189568

[DMM045641C50] Snyder A., Alsauskas Z. C., Leventhal J. S., Rosenstiel P. E., Gong P., Chan J. J. K., Barley K., He J. C., Klotman M. E., Ross M. J. et al. (2010). HIV-1 viral protein r induces ERK and caspase-8-dependent apoptosis in renal tubular epithelial cells. *AIDS* 24, 1107-1119. 10.1097/QAD.0b013e328337b0ab 20404718 PMC2860650

[DMM045641C51] Soler-García A. A., Rakhmanina N. Y., Mattison P. C. and Ray P. E. (2009). A urinary biomarker profile for children with HIV-associated renal diseases. *Kidney Int.* 76, 207-214. 10.1038/ki.2009.115 19357719 PMC2778294

[DMM045641C52] Strauss J., Abitbol C., Zilleruelo G., Scott G., Paredes A., Malaga S., Montané B., Mitchell C., Parks W. and Pardo V. (1989a). Renal disease in children with the acquired immunodeficiency syndrome. *N. Engl. J. Med.* 321, 625-630. 10.1056/NEJM198909073211001 2770791

[DMM045641C53] Strauss J., Abitbol C., Zilleruelo G., Scott G., Paredes A., Mitchell C., Parks W. and Pardo V. (1989b). HIV-associated nephropathy. *J. Pediatr.* 114, 336 10.1016/S0022-3476(89)80813-3 2915298

[DMM045641C54] Sunamoto M., Husain M., He J. C., Schwartz E. J. and Klotman P. E. (2003). Critical role for Nef in HIV-1-induced podocyte dedifferentiation. *Kidney Int.* 64, 1695-1701. 10.1046/j.1523-1755.2003.00283.x 14531802

[DMM045641C55] Tang P., Jerebtsova M., Przygodzki R. and Ray P. E. (2005). Fibroblast growth factor-2 increases the renal recruitment and attachment of HIV-infected mononuclear cells to renal tubular epithelial cells. *Pediatr. Nephrol.* 20, 1708-1716. 10.1007/s00467-005-2018-2 16133048

[DMM045641C56] Urbinati C., Nicoli S., Giacca M., David G., Fiorentini S., Caruso A., Alfano M., Cassetta L., Presta M. and Rusnati M. (2009). HIV-1 Tat and heparan sulfate proteoglycan interaction: a novel mechanism of lymphocyte adhesion and migration across the endothelium. *Blood* 114, 3335-3342. 10.1182/blood-2009-01-198945 19661268

[DMM045641C57] Woroniecki R. P., Ng D. K., Limou S., Winkler C. A., Reidy K. J., Mitsnefes M., Sampson M. G., Wong C. S., Warady B. A., Furth S. L. et al. (2016). Renal and cardiovascular morbidities associated with apol1 status among African-American and non-African-American children with focal segmental glomerulosclerosis. *Front. Pediatr.* 4, 122 10.3389/fped.2016.00122 27900314 PMC5110572

[DMM045641C58] Xie X., Colberg-Poley A. M., Das J. R., Li J., Zhang A., Tang P., Jerebtsova M., Gutkind J. S. and Ray P. E. (2014). The basic domain of HIV-tat transactivating protein is essential for its targeting to lipid rafts and regulating fibroblast growth factor-2 signaling in podocytes isolated from children with HIV-1-associated nephropathy. *J. Am. Soc. Nephrol.* 25, 1800-1813. 10.1681/ASN.2013070710 24578133 PMC4116058

[DMM045641C59] Ye X., Jerebtsova M., Liu X.-H., Li Z. and Ray P. E. (2002). Adenovirus-mediated gene transfer to renal glomeruli in rodents. *Kidney Int.* 61, S16-S23. 10.1046/j.1523-1755.2002.0610s1016.x 11841607

[DMM045641C60] Ye X., Jerebtsova M. and Ray P. E. (2000). Liver bypass significantly increases the transduction efficiency of recombinant adenoviral vectors in the lung, intestine, and kidney. *Hum. Gene. Ther.* 11, 621-627. 10.1089/10430340050015806 10724040

[DMM045641C61] Ye X., Liu X.-H., Li Z. and Ray P. E. (2001). Efficient gene transfer to rat renal glomeruli with recombinant adenoviral vectors. *Hum. Gene. Ther.* 12, 141-148. 10.1089/104303401750061203 11177551

[DMM045641C62] Zhong J., Zuo Y., Ma J., Fogo A. B., Jolicoeur P., Ichikawa I. and Matsusaka T. (2005). Expression of HIV-1 genes in podocytes alone can lead to the full spectrum of HIV-1-associated nephropathy. *Kidney Int.* 68, 1048-1060. 10.1111/j.1523-1755.2005.00497.x 16105035

[DMM045641C63] Zhu G., Nicolson A. G., Zheng X. X., Strom T. B. and Sukhatme V. P. (1997). Adenovirus-mediated beta-galactosidase gene delivery to the liver leads to protein deposition in kidney glomeruli. *Kidney In.* 52, 992-999. 10.1038/ki.1997.421 9328938

[DMM045641C64] Zuo Y., Matsusaka T., Zhong J., Ma J., Ma L.-J., Hanna Z., Jolicoeur P., Fogo A. B. and Ichikawa I. (2006). HIV-1 genes vpr and nef synergistically damage podocytes, leading to glomerulosclerosis. *J. Am. Soc. Nephrol.* 17, 2832-2843. 10.1681/ASN.2005080878 16988066

