# Supplementary material for: An HIV-Tat inducible mouse model system of childhood HIV-associated nephropathy
Source: Dis Model Mech. 2020 Oct 28;13(10):dmm045641. doi: 10.1242/dmm.045641 (PMC7648609; doi:10.1242/dmm.045641)
Supplement: Supplementary information [file dmm-13-045641-s1.pdf]

Supplemental Figure 1

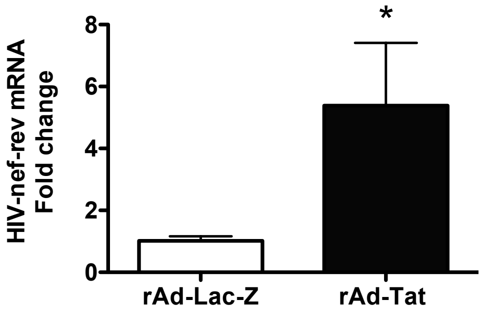

Figure S1. *rAd-Tat increased the expression of nef and rev in the kidneys of 7-day old HIV-Tg$_{26}$ mice.* The expression mRNA levels of *nef* and *rev* was quantified by real-time RT-PCR using the US and Art7 primers (Felser et al. 1991) as described in detail in the methods section. These primers predominately amplify a PCR product of 203 bp representing the nef cDNA and a 219/255 bp PCR product representing the *rev* cDNA in HIV-Tg$_{26}$ mice (Bruggeman et al. 1994). *p = 0.05, Mann Whitney t -test , n = 3 mice per group.

Disease Models & Mechanisms • Supplementary information

Supplemental Figure 2

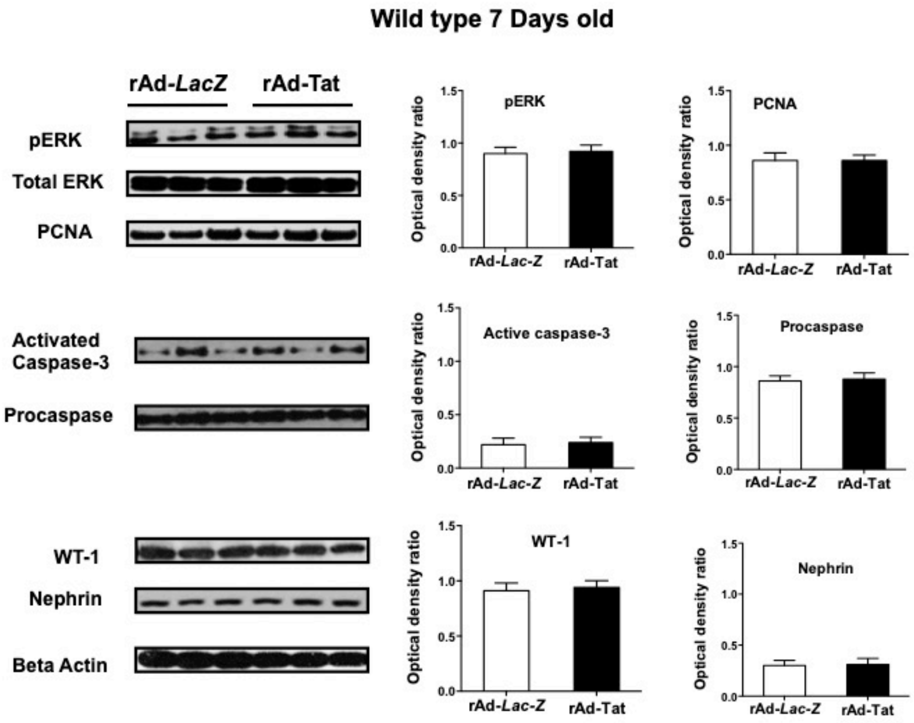

Figure S2. *rAd-Tat did not induce significant proliferative or anti-apoptotic changes in the kidney of 7 days old wild type mice.* The panels show representative results of the Western blot analysis for phospho-p44/42 MAPK (p-ERK), proliferating cell nuclear antigen (PCNA), activated caspase-3, procaspase, Wilms tumor 1 (WT1) and nephrin done with kidney homogenates derived from 7 days old wild type mice infected with rAd-*Tat* or rAd-*Lac-Z* vectors (n = 4 mice per group). The expression of PCNA, WT-1 and nephrin was quantified as a ratio of beta actin. The graphs show the results of the densitometry analysis and quantification of the results in optical density units (mean ± SEM), as described in the methods sections. Comparisons between groups were done by Mann Whitney t-test. All changes between groups were not statistically significant ($p > 0.05$).

Disease Models & Mechanisms • Supplementary information

Supplemental Figure 3

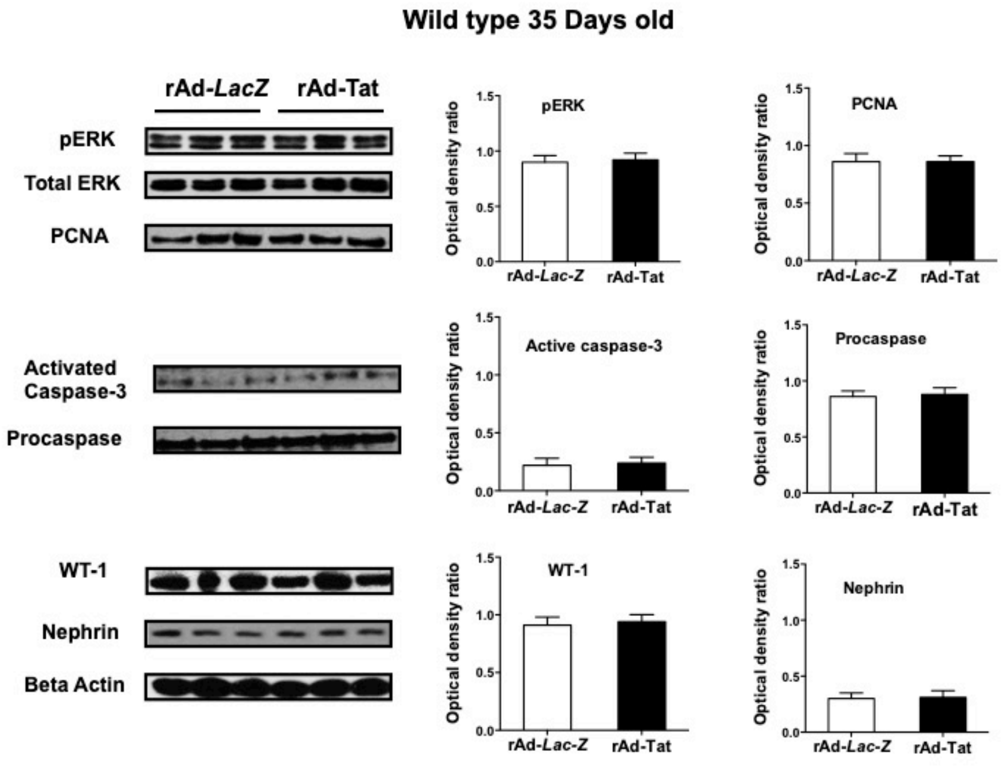

Figure S3. *rAd-Tat did not induce significant proliferative or anti-apoptotic changes in the kidney of 35 days wild type mice.* The panels show representative results of the Western blot analysis for phospho-p44/42 MAPK (p-ERK), proliferating cell nuclear antigen (PCNA), activated caspase-3, procaspase, Wilms tumor 1 (WT1) and nephrin done with kidney homogenates derived from 35 days old wild type mice infected with rAd-*Tat* or rAd-*LacZ* vectors (n = 4 mice per group). The expression of PCNA, WT-1 and nephrin was quantified as a ratio of beta actin. The graphs show the results of the densitometry analysis and quantification of the results in optical density units (mean ± SEM), as described in the methods sections. Comparisons between groups were done by Mann Whitney t-test. All changes between groups were not statistically significant ($p > 0.05$).

Disease Models & Mechanisms • Supplementary information

Supplemental Figure 4

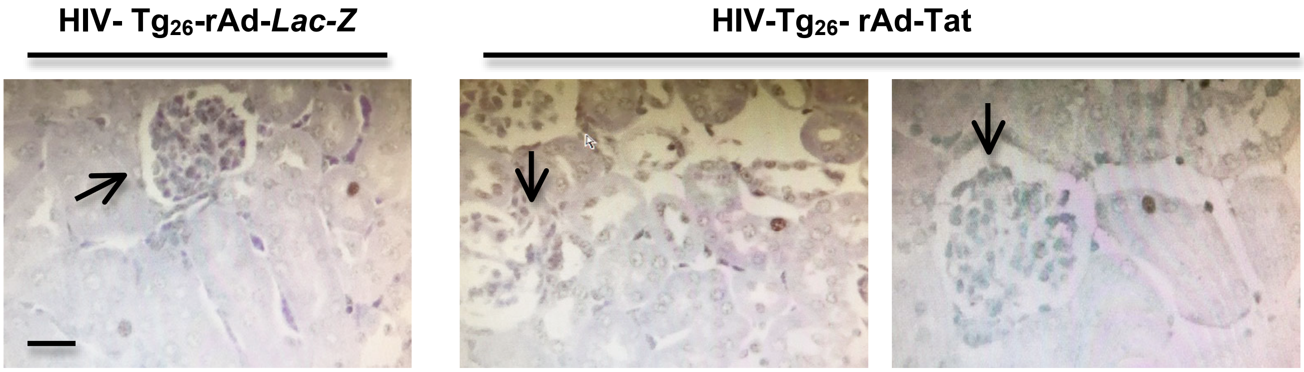

Figure S4. The panels show representative pictures of renal sections derived from HIV-Tg_{26} mice infected with rAd-*Lac-Z* and Tat vectors and stained with the TUNEL assay for apoptosis. Apoptotic cells were detected in renal tubules. The black arrows point to renal glomeruli. Scale bar, 30 μm.

Disease Models & Mechanisms • Supplementary information

Supplemental Figure 5

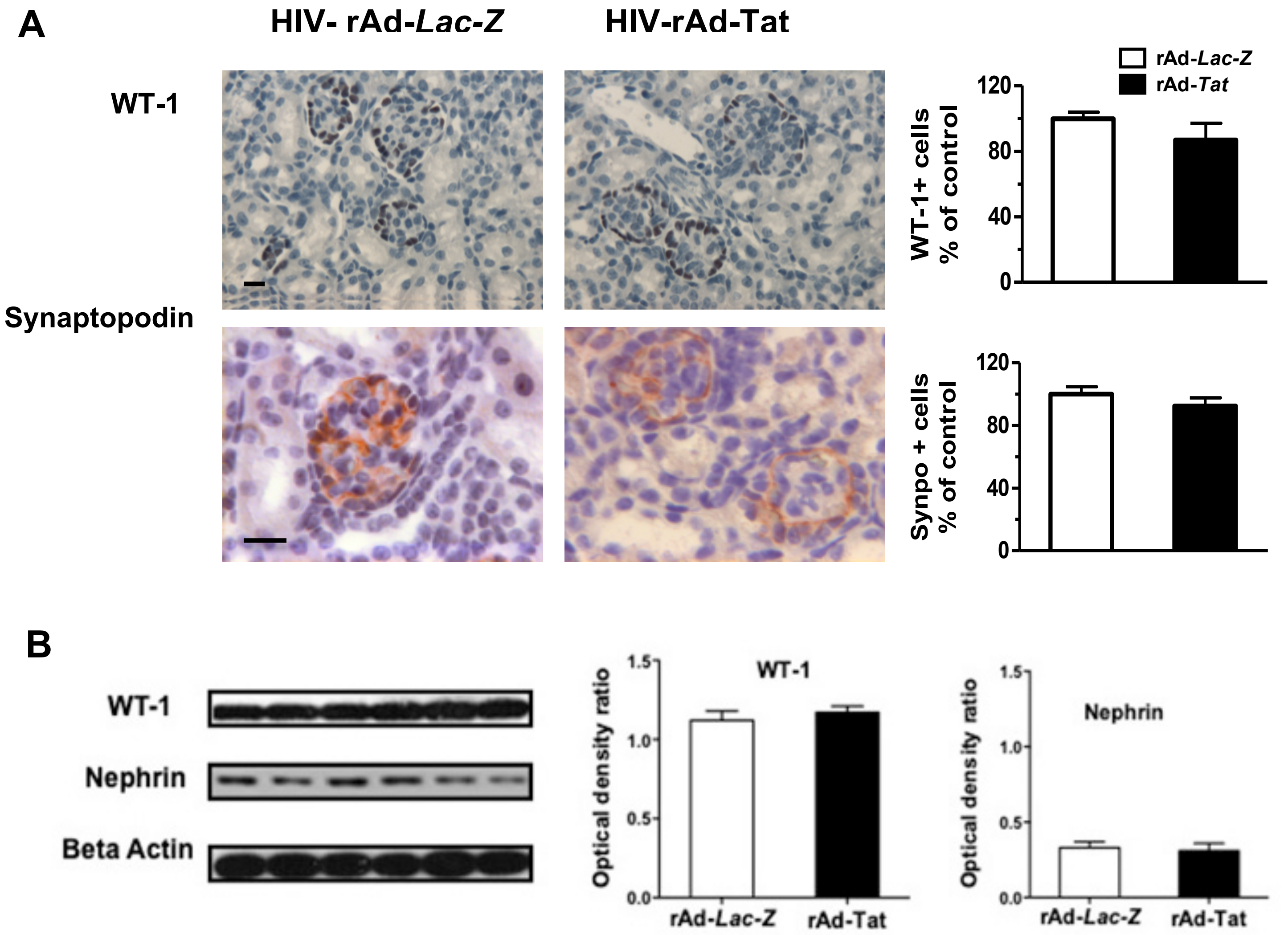

Figure S5. *rAd-Tat did not induce significant changes in the expression of WT-1, synaptopodin, and nephrin in 7 days old wild type mice.* **A.** The upper panels show a representative immunohistochemistry staining for WT-1 and synaptopodin (both red color) in renal sections harvested from 7 days old HIV-Tg_{26} mice infected with rAd-*Lac-Z* or *Tat* vectors. Scale bar, 20 μm. The graphs represent percentage changes in positive cells per field (mean ± SEM) relative to the controls ($p > 0.05$ by Mann Whitney t-test; n = 4 - 5 per group). **B.** The lower panels show representative Western blots for WT-

Disease Models & Mechanisms • Supplementary information

1 and Nephrin expressed as ratio of the beta actin protein. The graphs show the results of the densitometry analysis and quantification of the results in optical density units (mean ± SEM), as described in the methods sections ($p > 0.05$, by Mann Whitney t -test, n = 4 - 5 mice per group).

References

Felser, J. M., Klimkait, T. and Silver, J. (1989). A syncytia assay for human immunodeficiency virus type I (HIV-I) envelope protein and its use in studying HIV-I mutations. *Virology* **170,** 566-70.

Bruggeman, L. A., Thomson, M. M., Nelson, P. J., Kopp, J. B., Rappaport, J., Klotman, P. E. and Klotman, M. E. (1994). Patterns of HIV-1 mRNA expression in transgenic mice are tissue-dependent. *Virology* **202**, 940-8.
